# A Global View of Transcriptome Dynamics During Male Floral Bud Development in *Populus tomentosa*

**DOI:** 10.1038/s41598-017-18084-5

**Published:** 2018-01-15

**Authors:** Zhong Chen, Pian Rao, Xiaoyu Yang, Xiaoxing Su, Tianyun Zhao, Kai Gao, Xiong Yang, Xinmin An

**Affiliations:** 1 Key Laboratory of Silviculture and Conservation of the Ministry of Education, College of Forestry, Beijing Forestry University, Beijing, 100083 China; 2 National Engineering Laboratory for Tree Breeding, Key Laboratory of Genetics and Breeding in Forest Trees and Ornamental Plants of the Ministry of Education, College of Biological Sciences and Biotechnology, Beijing Forestry University, Beijing, 100083 China; 3 Berry Genomics Co., Ltd, Beijing, 100015 China; 4 Department of Forest Ecosystems and Society, Oregon State University, Corvallis, OR 97331 USA

## Abstract

To obtain a comprehensive overview of the dynamic transcriptome during male floral bud development in *Populus tomentosa*, high-throughput RNA-seq was conducted during eight flowering-related stages. Among the 109,212 *de novo* assembled unigenes, 6,959 were differentially expressed during the eight stages. The overrepresented classed of genes identified by Gene Ontology (GO) enrichment included ‘response to environmental stimuli’ and ‘plant-type spore development’. One-third of the differentially expressed genes were transcription factors (TFs). Several genes and gene families were analyzed in depth, including MADS-box TFs, *Squamosa promoter binding protein-like* family, receptor-like kinases, *FLOWERING LOCUS T*/*TERMINAL-FLOWER-LIKE 1* family, key genes involved in anther and tapetum development, as well as *LEAFY*, *WUSCHEL* and *CONSTANS*. The results provided new insights into the roles of these and other well known gene families during the annual flowering cycle. To explore the mechanisms regulating poplar flowering, a weighted gene co-expression network was constructed using 98 floral-related genes involved in flower meristem identity and flower development. Many modules of co-expressed genes and hub genes were identified, such as *APETALA1* and *HUA1*. This work provides many new insights on the annual flowering cycle in a perennial plant, and a major new resource for plant biology and biotechnology.

## Introduction

Flowering is one of the most important developmental events during the life cycle of angiosperms. In recent years, the molecular and genetic regulation of flower development has been studied in detail in *Arabidopsis* ^1^. Flowering is mainly controlled by six different endogenous (autonomous, gibberellin, and age) and environmental (photoperiod, temperature, and vernalization) pathways that together form a complex regulatory network^2^. All of these pathways are integrated through the transcriptional regulation of two floral pathway integrators, *FLOWERING LOCUS T* (*FT*) and *SUPPRESSOR OF OVEREXPRESSION OF CONSTANS 1* (*SOC1*). These activate the floral meristem identity genes, *LEAFY* (*LFY*) and *APETALA1* (*AP1*). *TERMINAL FLOWER 1* (*TFL1*, floral repressor) is involved in maintaining the vegetative and inflorescence meristems, by preventing the expression of *AP1* and *LFY*. *FT* and *SOC1* share an upstream regulator, *CONSTANS* (*CO*), which is a key component in the photoperiodic pathway, as well as *FLOWERING LOCUS C* (*FLC*), a potent floral repressor integrating autonomous and vernalization pathways^2^. Floral organ identity has been successfully described using the ABCDE model. Many ABCDE class genes are members of the MADS-box gene family and control the various processes of plant development^3^.

Unlike annuals, woody perennials have an extended juvenile phase. After they reach adulthood, flowering occurs annually and the life cycle of these trees alternates between growth and dormancy. Recent advances in plant genome studies have greatly facilitated investigation into the regulation of flowering and seasonal control of bud dormancy^4–7^. The *Populus* genome contains nearly all of the major orthologs regulating flowering in *Arabidopsis* ^8,9^. Gene expression analyses and functional studies in *Populus* have shown that flowering regulatory pathways are broadly conserved between *Arabidopsis* and *Populus* ^8^. However, the regulation of flowering in *Populus* also exhibits remarkably different, and sometimes unique, characteristics compared to annual plants. For example, *Populus PTLF* (the *LFY* ortholog in *P*. *trichocarpa*) was less effective at inducing early flowering^10^. The overexpression of *PTAP1-1* (*AP1* ortholog) does not induce early flowering in *Populus* ^11^. Unlike *AP1* in *Arabidopsis*, ectopic expression of *LAP1* (a tree ortholog of *AP1*) fails to induce early flowering in hybrid aspens; *LAP1* is instead involved in short-day (SD)-mediated seasonal growth cessation^12^. In transgenic poplars, *PtFT1* and *PtFT*2 (two very similar *Populus FT*-like paralogs) induce early flowering^13,14^. *PtFT1* is induced by winter temperatures and determines the onset of reproduction, while *PtFT2* is induced by long days and warm temperatures and then promotes vegetative growth^13,15,16^. *PtFT2* is down-regulated in response to SD, resulting in seasonal growth cessation^13,15^, and is up-regulated during chilling-induced dormancy release^15,16^. Unlike *CO* in *Arabidopsis*, the overexpression of poplar *CO1* and *CO2* does not alter normal reproductive onset^17^. The down-regulation of *PopCEN* (*CEN/TFL1* ortholog in *Populus*) by RNAi accelerates the onset of mature tree characteristics. *PopCEN1* also regulates axillary meristem identity and may play a role in maintaining the indeterminacy of the inflorescence apex^18^.

*P*. *tomentosa* Carr., widely grown in northern China, an important native commercial tree species used in urban green spaces, as well as in timber and pulp production. However, its extended juvenile phase presents a substantial obstacle to study and breeding. Due to the trichomes of catkins of female poplars in every spring, more male *P*. *tomentosa* are planted in northern China. But, from the aspect of wood accumulation, in general, biomass in male poplars is less than in female poplars. As we know, there is a banlance between vegetative growth and reproductive growth. Flower production requires large inputs of resources and energy, with negative impacts on vegetative growth. So, studying the flowering and flower development in male *P*. *tomentosa* is valuable. Additionally, the allergenic properties of poplar pollen in male floral buds are potential health hazards for allergic person^19,20^. Although a few flowering-related *Populus* genes have been discovered and studied^21^, a genome-wide study of the molecular basis of flower development in poplar is lacking. In-depth studies of the molecular basis of *Populus* flower development will contribute to a shortened breeding cycle for helping productivity and an understanding of the complex regulatory mechanisms controlling flowering in poplar.

Transcriptome sequencing using RNA-seq is a powerful tool for studying gene expression, defining gene putative function, and elucidating the molecular basis of key developmental processes^22^. In this study, we used paraffin-embedded tissues to examine the morphogenesis of male floral buds in *P*. *tomentosa*, and high-throughput sequencing to obtain global gene expression profiles during floral bud development in poplar. Based on extensive data analyses, we identified the MADS-box, *Squamosa promoter binding protein-like* family (*SPL*), receptor-like kinase (RLK) genes, *FT*/*TFL1*, and *CO*-like families, and a number of homologs of well-known floral genes. Furthermore, the construction of co-expression networks suggested a link between floral genes and the identified hub genes.

## Results and Discussion

### Floral bud development in *P*. *tomentosa*

**Figure 1:**
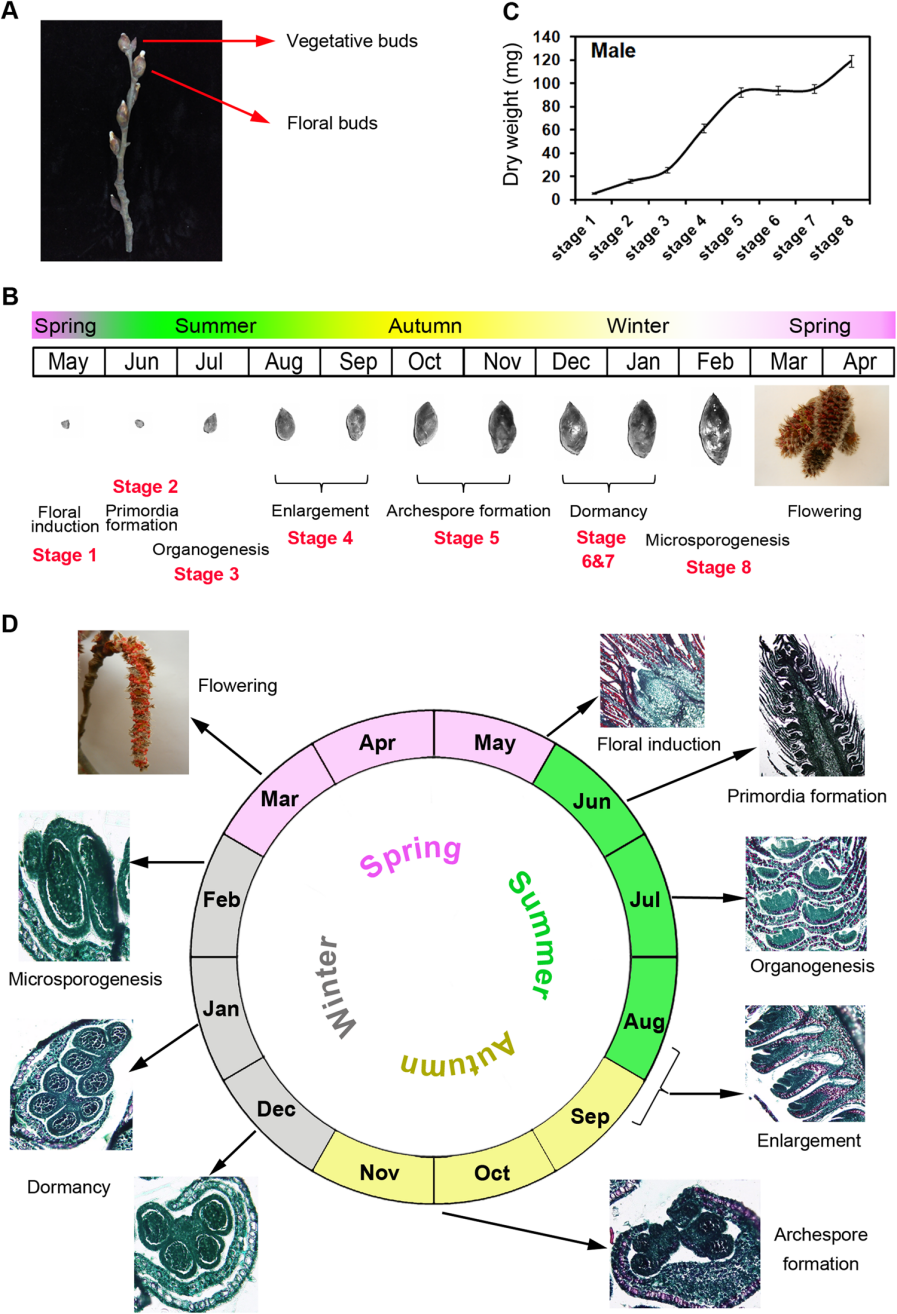
Schematic diagram of developmental stages responsible for the seasonal flowering of *Populus tomentosa*. (**A**) Axillary floral buds and vegetative buds. (**B**) Poplar male floral buds throughout the year. (**C**) Dry weight of *P*. *tomentosa* during flower development. (**D**) Poplar male floral buds at different stages throughout the year. Floral buds at stage 1: floral induction; stage 2: primordia formation; stage 3: organogenesis; stage 4: enlargement; stage 5: archespore formation; stages 6 and 7: dormancy; and stage 8: microsporogenesis.

Before the floral transcriptome study, we examined the developmental process of floral buds in a morphological analysis. The growth curves of the floral buds had a sigmoid shape. The dry weights of the buds increased progressively and rapidly from stage 1 to 4. During stages 5–7, floral buds increased continuously and then entered dormancy before again increasing (Fig. 1). These results showed that flower development was associated with bud size.

Floral buds embedded in paraffin were serially-sectioned to study their morphogenesis. Stage 1 buds are in the floral induction stage. The first morphological evidence of the transition from vegetative to reproductive growth in the axillary meristem is rapid elongation, bract formation, and the initiation of floral apices in the axils of each bract. Stage 2 buds are the smallest flower buds that can be distinguished from vegetative buds. During this stage, centripetal bract formation continues, the floral meristems are surrounded by the cup-like, reduced perianth developing in the axils of the bracts, and the bracts extend to completely enclose the inflorescence. In stage 3 buds, rounded stamen primordia emerge from the meristem, with organogenesis proceeding centrifugally. Tetrasporangiate anthers form and are surrounded by the reduced perianth cup in the axils of mature bracts. Each anther typically contains four microsporangia, with each microsporangium forming a locule. Microspore mother cells develop initially in each of the four locules. The microsporocytes are arranged compactly within the locule, at which stage the male buds over-winter. In February of the next year, the inner-wall layer of the microsporangium matures, with the tapetum serving as a nutritive layer. The microsporocytes form tetrads after meiosis. As the spores mature, the tapetal cells disintegrate and each microspore divides mitotically to form a pollen grain. After microsporogenesis, the mature pollen grains are released from the dehisced anther (Fig. 1D).

### Global view of the *P*. *tomentosa* floral transcriptome

A mixed-reads pool obtained from all stages of buds generated 296,623 transcripts >200 bp in length and 109,212 unigenes with an average length of 640.77 bp. The N50 for transcripts and unigenes was 1,928 bp and 873 bp, respectively (Table 1). The size distribution of the transcripts and unigenes, and the results of the sequence similarity analysis against various databases, are showned in Fig. S1 and Table 2. Most of the annotated unigenes had highest similarity to *P*. *trichocarpa* (78.40%) (Fig. S2A).

**Table 1:** Summary of the floral transcriptome of *P*. *tomentosa*.

	Transcript	Unigene
Total nucleotide length	332,144,927 bp	69,979,229 bp
Total number of contigs	296,623	109,212
Max. length	16,966 bp	16,832 bp
Min. length	201 bp	201 bp
Average length	1119.75 bp	640.77 bp
N50	1928 bp	873 bp
N90	458 bp	278 bp
GC%	60.31	61.10

**Table 2:** Annotation of the unigenes in *P*. *tomentosa*.

Sequence database	Number of annotated unigene sequences	Percentage of annotated unigene sequences
Total unigenes	109,212	100
Nr	35,140	32.18
KEGG	35,591	32.59
GO	16,841	15.42
COG	27,040	24.76

Of the 35,140 Nr hits, 27,040 sequences had a COG classification (Table 2). Among the 25 COG categories, ‘General function prediction only’, ‘Posttranslational modification’, and ‘Signal transduction mechanisms’ were the largest groups (Fig. S2B). Among all 109,212 unigenes, 16,841 were successfully annotated with GO terms (Table 2). All leading GO terms at level 2 could be categorized into 52 groups (Fig. S2C), indicating an association between the identified genes and various biological processes. In addition to cellular and metabolic processes, many genes were assigned to ‘biological regulation’, ‘response to stimulus’, ‘pigmentation’, and ‘developmental process’. The prominence of ‘binding’ suggested an important role for TFs during flower development in *P*. *tomentosa*.

The relationships between the floral buds were investigated in a PCA of the whole-gene expression dataset. Stages 1–4 clustered closely, as did stages 5–7 (Fig. S3A). This indicated that similar transcriptional programs, albeit with distinct differences as well, were active within the floral buds at different stages. A count of the number of genes expressed at different levels in the eight samples showed medium-level expression (2 ≤ FPKM < 10) by the largest portion of transcripts, followed by high-level expression (FPKM ≥ 10). The proportions of genes at the three expression levels (high, medium, and low) were similar in all stages (Fig. S3B).

### Changes in transcriptome profiles during flower development

**Figure 2:**
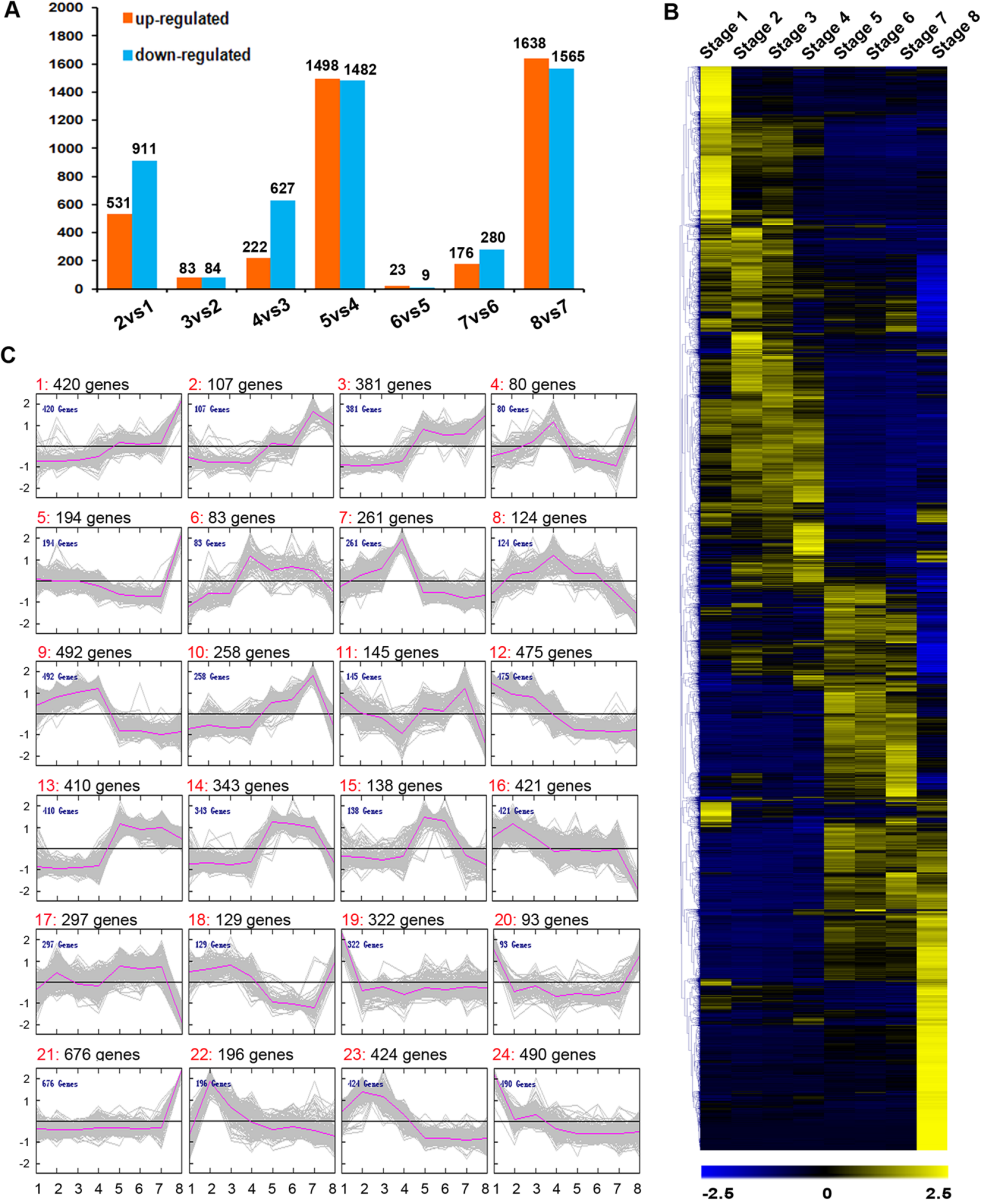
Gene expression dynamics during the different stages of flower development in *P*. *tomentosa*. (**A**) The number of up- and down-regulated genes during the various stages of flower development with respect to the preceding stage. (**B**) Hierarchical cluster analysis. (**C**) K-means clustering of the DEGs during the eight developmental stages. Black numbers on the top are the number of genes for each cluster; the red numbers are the cluster labels.

Candidate genes involved in *P*. *tomentosa* flower development were identified in differential expression analyses between consecutive time points, resulting in the identification of 6,959 DEGs during flower development. Of these, 531, 83, 222, 1,498, 23, 176 and 1,638 genes were significantly up-regulated and 911, 84, 627, 1,482, 9, 280, 1,565 genes were significantly down-regulated in stages 2–8, respectively, when compared to the preceding stage (Fig. 2A). Notably, a particularly large number of genes was up- or down-regulated in stage 5 versus stage 4, and in stage 8 versus stage 7, which indicated a large shift in the transcriptional programs. This result was consistent with the PCA (Fig. S3A). Hierarchical and K-means clustering of all DEGs are shown in Fig. 2B,C.

### GO enrichment

The major functional categories during floral development were identified in a GO enrichment analysis of 6,959 DEGs. Many significantly enriched GO terms were related to floral development, organ development, and secondary metabolite synthesis. In biological process, ‘response to abiotic stimulus’, ‘plant-type spore development’, and ‘cellular macromolecular complex subunit organization’ were highly represented. For molecular function, GO terms related to structural molecule activity and various aspects of ‘oxidoreductase activity’ were significantly enriched. In the category ‘cellular component’, significantly enriched genes were associated with organelles, membrane, and the cell wall (Fig. S4).

The enriched biological process GO terms of the up-regulated genes were analyzed for each of two consecutive time points. Although many of the enriched GO terms were common, a few were unique in different sets of genes. For example, the GO terms related to lipid and fatty acid biosynthesis were enriched in stages 2 and 3, RNA splicing in stage 3, amino acid catabolic processes in stage 4, DNA repair and signaling in stage 5, localization in stages 6 and 7, and defense responses and cell proliferation in stage 8 (Fig. S5).

### Transcription factors

TFs are the key regulatory proteins, mediate the transcriptional regulation by binding to specific motifs in the promoter of target genes. TFs are known to play a significant role in floral development. Among the 6,959 DEGs, approximately one-third (2,231) of the DEGs were TFs. These genes fell into 54 diverse categories, covering nearly all TF families, with ERF, MYB, bHLH, NAC, B3, bZIP, and MIKC being the most highly represented. Hierarchical and K-means clustering analyses depicting the differential expression profiles of the TF genes during floral development are presented in Fig. S6.

### Type II MADS genes

Many of the key regulatory genes involved in floral development and flowering time are members of the MADS-box family of TFs. In plants, MADS-box family proteins are divided into types I and II, with the latter further classified into MIKC^C^ and MIKC^*^.

In *Arabidopsis*, the MIKC^*^ group is involved in pollen maturation^23^. In peach, the expression of MIKC^*^ genes during floral development is higher in pollen than in other tissues^24^. In apple, MIKC^*^ genes are highly expressed at later stages of flower development^25^. Our results showed higher expression of close orthologs of these genes in *P*. *tomentosa* (*PtAGL65*.*2*, *PtAGL65*.3, and *PtAGLS4*) at later stages (Fig. S7), suggesting that MIKC^*^ genes may be involved in pollen development in this species.

**Figure 3:**
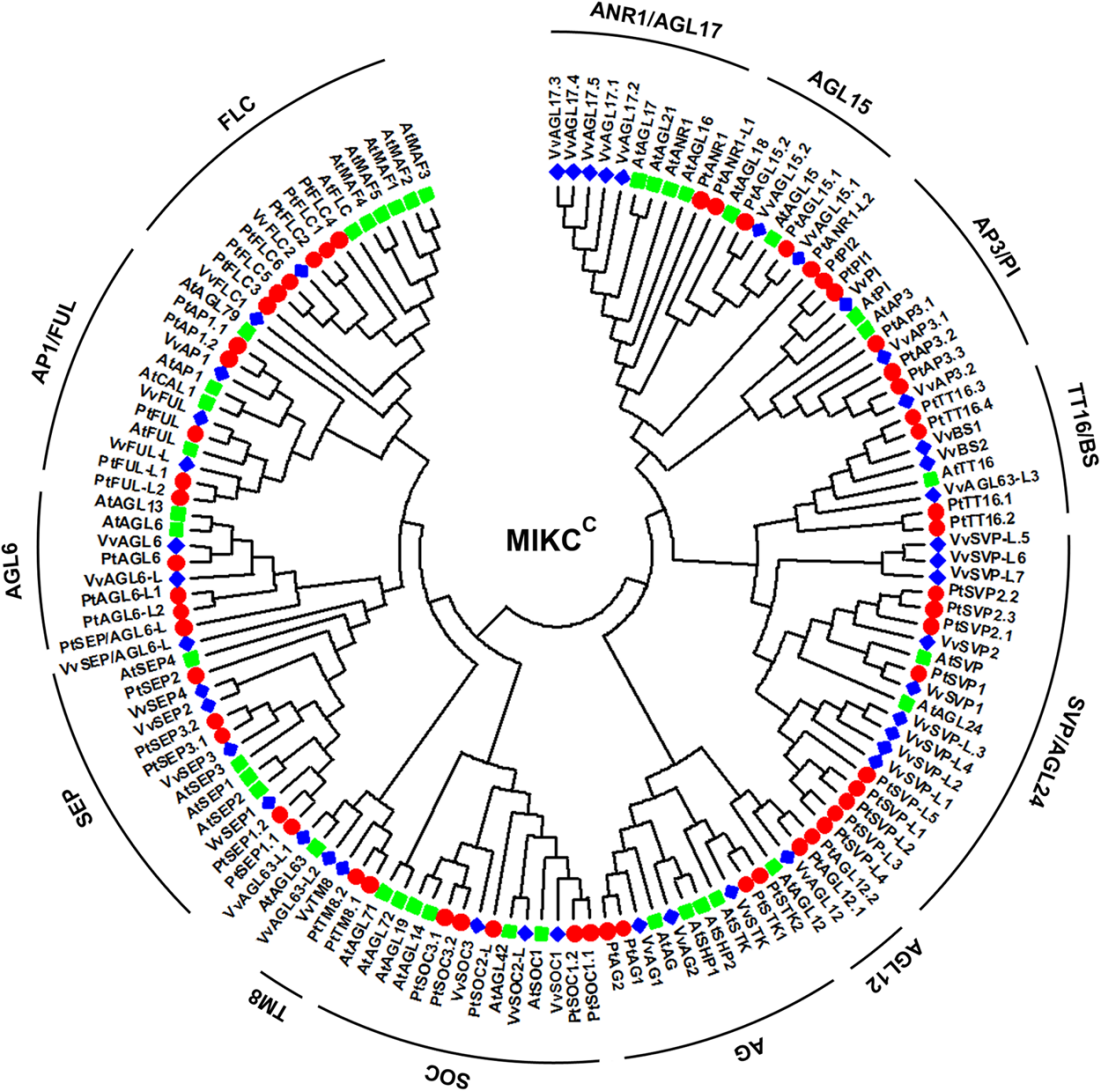
Phylogenetic tree of the MIKC^c^ MADS-box proteins in poplar, *Arabidopsis*, and grapevine. The tree was generated after sequence alignment using the neighbor-joining method. Bootstrap values from 1,000 replicates were used to assess the robustness of the tree.

**Figure 4:**
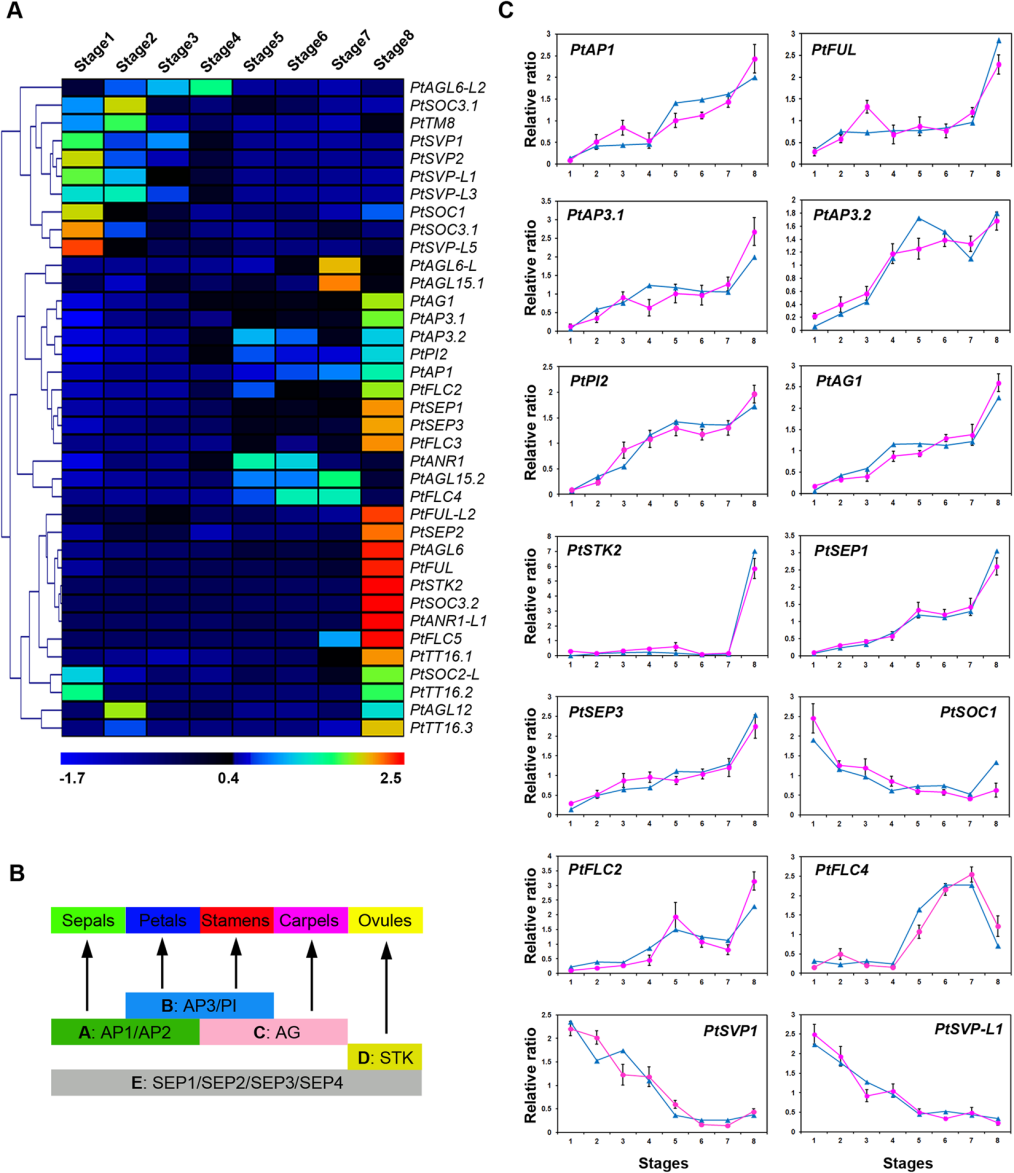
Expression profiles of MIKC^c^ MADS-box genes in the floral transcriptome of *P*. *tomentosa*, and the identification of those genes by RT-qPCR. (**A**) Hierarchical cluster analysis of the identified MIKC^c^ MADS-box genes in the floral transcriptome of *P*. *tomentosa*. (**B**) Schematic representation of the classical ABCDE model of flower development. Class A genes specify sepals; class A and B genes, petals; class B and C genes, stamens; class C genes, carpels; class D genes are specifically expressed in the ovules, and class E genes have partially redundant functions in different floral organs. (**C**) RT-qPCR validation of the expression levles of MIKC^c^ MADS-box genes in floral buds. The blue and pink lines were derived from RNA-seq and RT-qPCR data, respectively. The values are means ± SD of three replicates.

To determine the phylogenetic relationships among poplar MADS-box proteins, and then to group the respective genes within established subfamilies, we generated phylogenetic trees containing the full-length proteins from *Arabidopsis*, grapevine, and poplar. The MIKC^C^ genes were divided into 13 subfamilies (Fig. 3). In this transcriptome, 37 MIKC^C^ genes were identified. Their expression profiles in floral buds were compared by hierarchical clustering together with RT-qPCR to quantify the expression of MADS-box genes (Fig. 4). As a general rule, closely related genes within subfamilies display conserved expression patterns, although the expression levels of specific members may change during different stages. Hierarchical clustering showed that the MIKC^C^ genes formed three clusters (Fig. 4A).

### Floral homeotic genes

MIKC^C^ genes from the ABCDE model function as floral homeotic genes in *Arabidopsis* and other plants, where they specify floral meristem and floral organ identity^26^ (Fig. 4B).

The *Arabidopsis* A-class gene *AP*1 contributes to specifying floral meristem identity and participates in the development of sepals and petals^26^. In the present study, *PtAP1* expression increased gradually during floral bud development (Fig. 4A,C). A similar expression pattern was reported for *C*. *azalea* floral buds^27^. In other species of *Populus*, *PTAP1-1* and *PTAP1-*2 are expressed throughout the initiation of floral meristems^8^. In another dioecious plant of Salicaceae, willow, *SAP1* is strongly expressed in all layers of the inflorescence meristem, in the developing flowers along the flanks of the inflorescence meristem, in the bract primordial, in young bracts, and in floral meristems^28^. In apple, *AP1* orthologs are expressed at high levels during flower and fruit development^25^. The overexpression of apple *AP1* orthologs causes early flowering in both tobacco and *Arabidopsis* ^29,30^, whereas the overexpression of *PTAP1-1* does not induce early flowering in *Populus* ^11^. A recent study showed that, in hybrid aspen, *LAP1* mediates photoperiodic control of seasonal growth cessation, acting downstream of the *CO*/*FT* module. Down-regulation of *LAP1* is required for SD-mediated growth cessation. In contrast with *AP1* targets in flowering, *LAP1* connects the *CO*/*FT* module with the regulation of AINTEGUMENTA-like 1, which plays a role in SD-mediated growth cessation^12^.

B-class function is defined by *AP*3 and *PI*; these genes specify the identity of petals and stamens^26^. In the present study, *PtAP3*.*1*, *PtAP3*.2, and *PtPI2* increased during flower development, reaching a peak during stage 8 (Fig. 4A,C). A similar pattern of *PtAP3* and *PdPI* expression was observed by An *et al*.^19^ and Zhang *et al*.^31^, respectively. In *Eucalyptus grandis*, *EgPI1*-*3* are also up-regulated during late compared to early flower development^7^. During microsporogenesis (stage 8), high-level expression of *PtAP3* and *PtPI* indicated the close relationship of these genes to pollen maturation. *AP3*/*PI* genes are highly expressed in peach pollen^24^ and in the floral tissues of Japanese apricot^32^ and apple^25^. In black cottonwood, the *AP3* ortholog is initially expressed in the inner whorl of both male and female floral meristems and maintained in the stamen primordia as the reproductive primordia begin to form^33^. The similar spatial expression pattern of *AP3*/*PI* clade genes in poplar, peach, Japanese apricot, and apple is consistent with their functional conservation in perennial woody plants.

The *AG* subfamily includes C- and D-class genes. The C-function is defined by *AG*, which specifies the stamen and carpel, and the D-function by *SHP1*&2 and *STK*, which shape the identity of the ovules^26^. In the RNA-seq data, *PtAG1* was expressed first at low levels during early stages, and then at higher levels, whereas *PtSTK2* was exclusively expressed during stage 8, suggesting its role in later developmental events (Fig. 4A,C). Similarly, the levels of *EgAG* and *EgSTK* expression increased during floral bud development in *Eucalyptus* ^7^. In *P*. *trichocarpa*, *PTAG1*&*2* are expressed in the inner whorl of male and female flowers both before and after reproductive primordia emerge, indicating their function conservation in specifying male and female reproductive identity^34^. The rapid up-regulation of *PtSTK2* during microsporogenesis suggested its importance in specifying male reproductive identity. Interestingly, in our data, we did not detect any homologs of *Arabidopsis SHP1*&2, as also reported for grapevine^35^. In other perennial trees, such as apple, peach, and Japanese apricot, *AG* homologs are highly expressed during flower and fruit development^24,25,32^.

*SEP* genes provide the E function and play redundant roles in floral organ and meristem identity in four whorls^26^. Tomato and strawberry *SEP* orthologs contribute to fruit development^36,37^. In this study, four *SEP* orthologs were identified. The expression of *PtSEP1* and *PtSEP3* increased gradually during floral bud development, indicating their increasingly important role from floral induction to organ development (Fig. 4A,C). Similarly, in *P*. *tremuloides*, the *SEP*-class genes *PTM3*/4 and 6 are expressed in all stages of male and female floral development, predominantly in the inner sexual whorl, within developing ovules of female flowers, and in the anther primordia of male flowers^38^. In apple, grape, and apricot, the high-level expression of *SEP* orthologs is maintained throughout development, from flower initiation to fruit development^25,32,35^.

In previous study, *PtAP3* was not restricted to floral buds but was expressed at similar levels in the vegetative tissues of *P*. *tomentosa* ^19^. In addition to male floral buds and inflorescences of *P*. *deltoides*, *PdPI* is expressed in roots, female inflorescences, immature xylem, leaves, and apical buds^31^. In *P*. *trichocarpa*, the C-class gene *PTAG* is consistently expressed in vegetative tissues^34^. Expression of the *P*. *tremuloides* E-class genes *PTM3*&4 occurs in terminal buds, young leaves, and stems^38^. The broad expression of poplar BCE-class genes suggests their additional roles in vegetative development and is consistent with the long evolutionary history of angiosperms^39^.

### *SOC* subfamily

In *Arabidopsis*, *SOC*1 functions as a flowering promoter, integrating signals from multiple pathways, together with AGL24, which activates downstream targets, such as the flowering promoter *FT* and the flower meristem identity genes *AP*1 and *LFY* ^2^. In our study, *PtSOC*1 and *PtSOC3*.*1* were located to cluster 1, and *PtSOC2-L* to cluster 3. Cluster 1 transcripts were highly expressed in floral buds during the initiation and proliferation of inflorescence primordia (stages 1 and 2). *PtSOC1* and *PtSOC3*.*1* expression was highest in stage 1 (floral induction) and then decreased (except for the later increase of *PtSOC1*), which suggested that these genes play a crucial role in flowering induction (Fig. 4A,C). Similarly, in grape, the expression of *VvSOC1*.*1* decreased during flower meristem initiation and flower development^35^. In apple, *MdSOC1* expression gradually declined during flower induction^40^. In *E*. *grandis*, nearly half of the type II MADS-box genes belong to the *SOC* subfamily; its expansion has been attributed to tandem duplications^7^. By contrast, in our study, the *P*. *tomentosa* transcriptome contained only four members of the *SOC* family. Previous study similarly reported only five members in the *SOC* subfamily of *P*. *trichocarpa* ^7^. Thus, in poplar, the *SOC* subfamily did not undergo huge expansion that occurred in *E*. *grandis*.

### *FLC*-like and *SVP*/*AGL24* subfamilies

In *Arabidopsis*, the MIKC^c^ genes regulating flowering transition mainly belong to the *FLC*, *SVP*, and *SOC* subfamilies. *FLC* act as an important floral repressor active in leaves and the apical meristem. Its expression is down-regulated after long-term cold during vernalization. In addition, *FLC* represses *SOC1* and *FT* and interacts with *SVP* to repress of *SOC1* and *FT* ^26^. We identified four *FLC*-like genes (*PtFLC2*–5), all of which were mainly expressed during later stages. Of these, *PtFLC*2 and *PtFLC4* showed opposing expression pattern (Fig. 4A,C), as reported for two *FLC* homologs (*VvFLC1*&*2*) in the buds of grapevine^6^. *PtFLC2* expression decreased in response to chilling during winter dormancy and increased after dormancy release (Fig. 4). Similar patterns of *VvFLC1* expression in grape^6^, and of *PEP1* (*FLC* homolog) in *Arabis alpina* ^41^, have been described. By contrast, the expression of *PtFLC4* increased during dormancy and declined when growth resumed after the cold period. Similar results were reported for the *FLC*-like genes in apple^25^, *Arabidopsis* ^42^, and grape^6^. In *P*. *tomentosa*, divergence between the sequences of *FLC*-like genes and those of *Arabidopsis* and their different expression patterns may explain their functional divergence. Previous study in poplar showed that *FLC*-like assumed different functions in apical bud development and dormancy^43^.

In *Arabidopsis*, the *SVP*/*AGL24* subfamily contains only two members: *SVP* acts as a floral repressor, through the negative regulation of *FT*, while *AGL24* promotes flowering by positively regulating *LFY* ^26^. We identified five orthologs of *SVP*/*AGL24* from the RNA-seq data. *PtSVP* expression was high during floral induction and initiation, then decreased gradually, without significant changes during dormancy (Fig. 4A,C), what would not justify their role in dormancy. In *Eucalyptus*, *EgSVP1* was down-regulated in late versus early flower development^7^. Expression of the *SVP* ortholog in *Camellia azalea* was also reduced during floral bud development^27^. In *Populus*, low temperatures and long-days are required to break bud dormancy and they determine the specific flowering time^16^. In peach and leafy spurge, *SVP* orthologs, as dormancy-associated MADS-box genes (*DAM*),are important in growth cessation, bud set, and seasonal vegetative and floral bud dormancy^44,45^. Our data showed a weak reduction in *PtSVP-L1* expression during dormancy release (Fig. 4A,C). Dormancy induction signals activate *DAM* expression, which then decreases with dormancy release in leafy spurge^45^, Japanese apricot^46^, raspberry^47^, and apple^25^.

In summary, MADS-box gene family play important roles in floral development in poplar. Here, MADS-box homologs were identified and their expression patterns at eight stages were studied. According to these results, their potential functions were speculated, but the exact function still needs further studies by genetic transformation, such as overexpression or silence (RNAi and CRISPR-Cas9). Previously, Song *et al*.^48^ also explored the floral development in *P*. *tomentosa* using transcriptome, as well as phytohormone and DNA methylation analysis. However, they focused on the differences between male and female poplars during floral development at transcriptional level. Relatively limited candidate genes (24 genes) were chosen for confirming their expression pattern during floral development in males and females. Although these genes might play important roles during flower development, they mainly involved in phytohormone synthesis and metabolism, but few genes were involved in tranditional floral pathways. Floral development is a dynamic developmental process and regulated by many related genes in complex network. Our study aimed to generate a global view of genome-wide transcriptome dynamics during floral bud development in *P*. *tomentosa*. Besides MADS-box genes, numerous other genes associated with floral development, such as *SPL* gene family and *RLK* genes etc., were identified and their expression patterns were also analyzed. All of these will be necessary and important to elucidate molecular basis of flower development in *Populus*.

### *SPL* gene family

*SPL* family play crucial roles in plant growth and development processes, such as phase transition, flowering, fruit development and architecture^6^. In *Arabidopsis*, the *SPL* family has 16 members. *AtSPL1*, 7, 12, 14 and 16 are expressed constitutively, while the remaining genes are highly expressed in flowers^49^. Previous study in poplar identified 28 *SPL* family members^50^.

**Figure 5:**
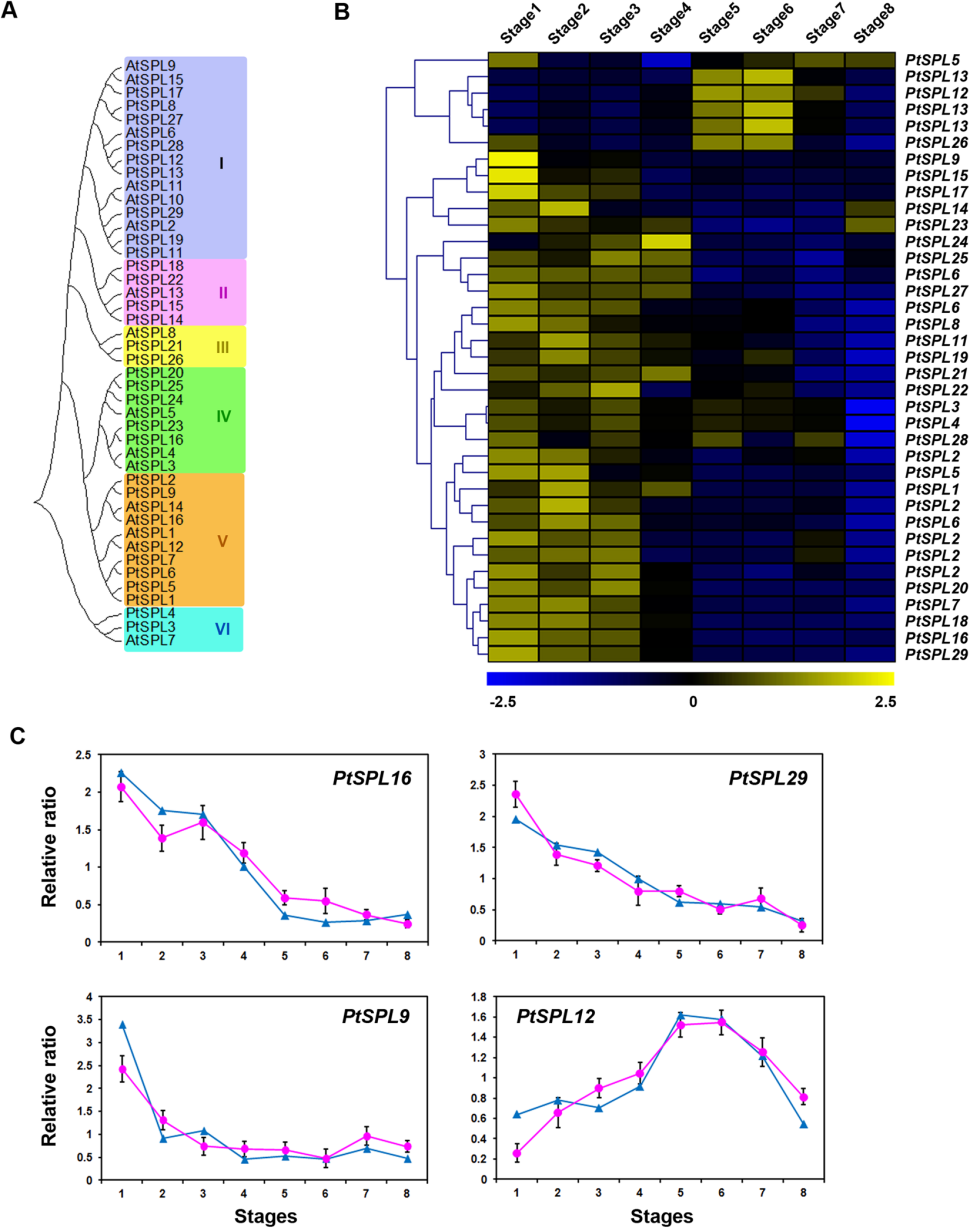
Expression profiles of the *squamosa promoter binding protein-like* family (*SPL*) gene family in the floral transcriptome and their RT-qPCR validation. (**A**) Phylogenetic tree of SPL family proteins in poplar and *Arabidopsis*. The tree was generated after sequence alignment using the neighbor-joining method. Bootstrap values from 1,000 replicates were used to assess the robustness of the tree. (**B**) Hierarchical cluster analysis of the identified *SPL* genes in the floral transcriptome of *P*. *tomentosa*. (**C**) RT-qPCR validation of the *SPL* gene expression levels in floral buds. The blue and pink lines were derived from the RNA-seq and RT-qPCR data, respectively. Values are means ± SD.

Ten of the sixteen *AtSPL*s (2–6, 9–11, 13 and 15) are post-transcriptionally regulated by the miR156 family, resulting in the incorporation of endogenous age/development signals into vegetative phase transition and flowering^51,52^. This regulatory mechanism is conserved in woody perennials^6^. *AtSPL3*–5 contain sequences complementary to miR156 in the 3′ UTR; all of those genes promote the juvenile-to-adult phase transition and flowering^53^. In our study, their orthologs, *PtSPL16*, 20, and 23–25,were highly expressed in the early stages of floral buds (Fig. 5). *AtSPL2*, 10, and 11 regulate the morphological changes in cauline leaves and flowers during the reproductive phase^54^. Their orthologs, *PtSPL11*, 19, and 29, decreased during floral bud development (Fig. 5). *AtSPL9* and 15 act redundantly in controlling the juvenile-to-adult phase transition and the leaf initiation rate^55^. Orthologs *PtSPL8*, 17, and 27 were located close together in the phylogenetic tree (Fig. 5A).

Six *AtSPL*s are not targets of miR156/7 in *Arabidopsis*. Among them, *AtSPL8* regulates pollen sac development^56^ and male fertility^57^. The expression of *PtSPL26* (*AtSPL8* ortholog) is restricted to the dormancy period (Fig. 5B). *AtSPL14* regulates plant architecture and represses both the vegetative phase transition and flowering^58^. In the present study, the transcription of *PtSPL9*, a homolog of *AtSPL14*, was highest during the floral induction stage (Fig. 5).

*PtSPL12*&13 (*AtSPL6* homologs) expression was highest during dormancy (Fig. 5) and the pattern was similar to that of *PtFLC4*; these *SPL* genes may be involved in dormancy maintenance. The expression of *PtSPL12*&13 decreased during dormancy (Fig. 5). *SPL*-like 3&6 have also been detected during the dormancy of other *Populus* species, with increases in *SPL6*-like and decreases in *SPL3*-like as dormancy progressed^59^. Further studies are needed to elucidate the roles of the *SPL*s in flowering and dormancy.

### *RLK* genes

RLK play important roles in flower development and reproduction, including tapetum development and microspore maturation^60^, male-female interactions^61^, as well as pollen tube reception^62^. In this study, we identified 216 unigenes annotated as RLK. Their hierarchical clustering is shown in Fig. S8. The *Arabidopsis ERECTA* (*ER*) family receptor kinases *ER-LIKE1* (*ERL1*), and *ERL*2 regulate inflorescence architecture, floral meristem organization, and floral organ identity^63^. In our study, *PtERL1* and *PtERL4* (comp650512_c0 and comp484228_c0) were expressed consistently during floral bud development (Fig. S8), indicating their roles in flower development. *Arabidopsis* somatic embryogenesis receptor-like kinases 1&2 (*SERK1*&2) are essential for tapetum development and microspore maturation^60^. In our study, *PtSERK1* and *PtSERK*2 were down-regulated during floral bud development, while the transcripts of comp619813_c0 and comp636715_c0 (two other homologs of *AtSERK 1*&2) reached their highest level during stage 8 (Fig. S8), indicative of their different functions during floral organ development.

### Other well-known genes

Some genes specifically involved in anther or tapetum development were clearly up-regulated during stage 8 but were undetected or expressed at extremely low levels during earlier stages. These genes included *AMS*, *CYP703A*2, *TDF1*, and *ACOS5* (Fig. S9) and their expression coincided with active anther and stamen development. Similar pattern of up-regulation was observed in the late-stage of floral buds in *Eucalyptus* ^7^. In other species, these genes were shown to be associated with later stages of tapetum and pollen development, such as pollen wall formation^64–66^. The transcripts of other genes required for tapetum development, such as *EMS1* and *TPD1*, reached their highest level during stage 5 and 6 (Fig. S9). In addition, genes involved in pollen tube growth, pollen hydration, and callose degradation during tetrad dissolution, such as *POE1*, *SKU5*-like, *PME*, *OLE*, and *βGLU*, were differentially expressed during floral bud development^67^ (Fig. S9A).

**Figure 6:**
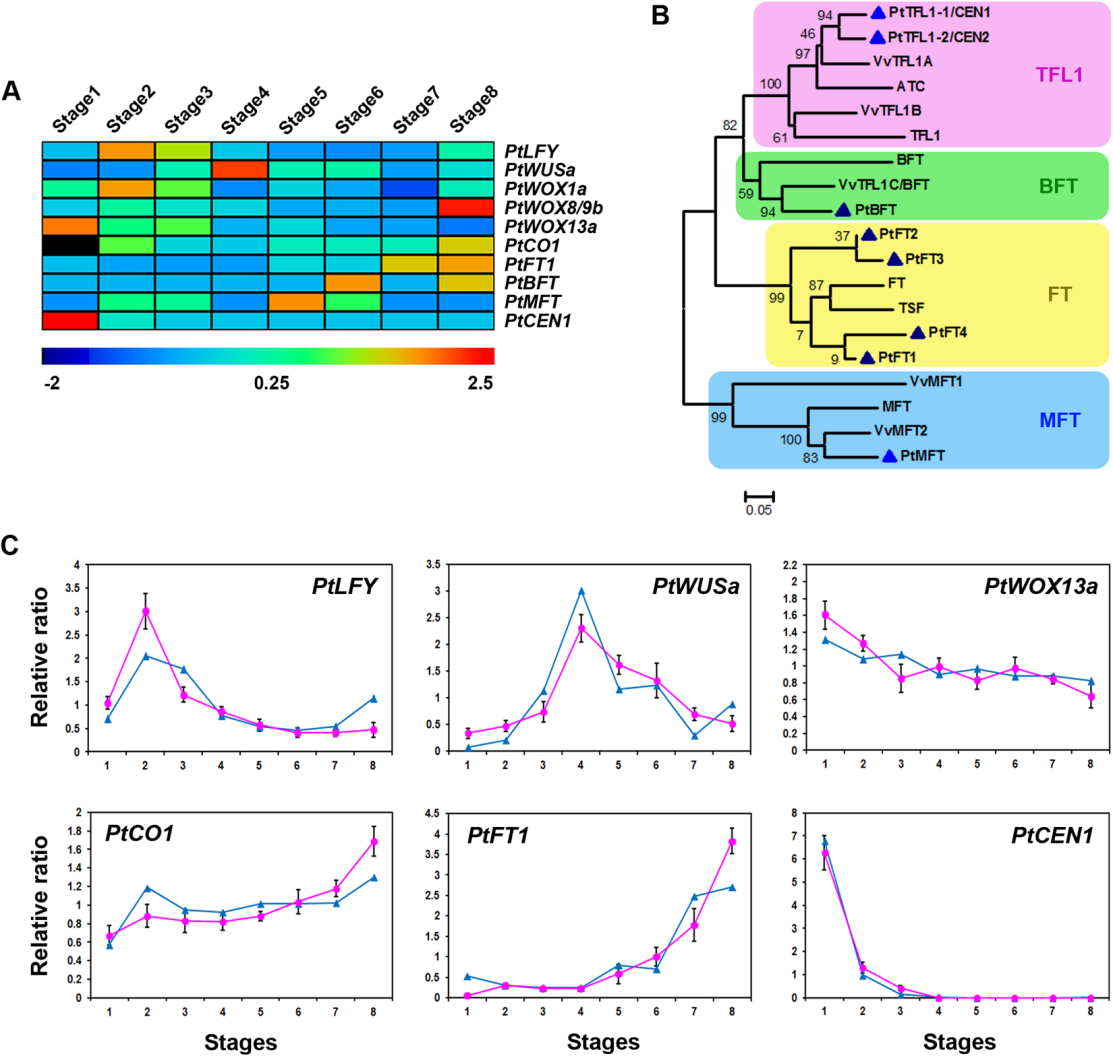
Expression profiles of several well-known floral-related genes and their RT-qPCR validation. (**A**) Heat map of gene expression. (**B**) Phylogenetic tree of the poplar, *Arabidopsis*, and grape *FT*/*TFL1* gene family containing phosphatidylethanolamine-binding proteins (PEBPs), obtained using the neighbor-joining method. The unit for the scale bar indicates branch lengths (0.05 substitutions per site). (**C**) RT-qPCR validation of the expression profiles obtained by RNA-seq. The values are means ± SD.

*PtLFY* was first up-regulated, then down-regulated, during floral bud development but increased during stage 8 (Fig. 6). In *Arabidopsis*, *LFY* is a floral meristem identity gene, regulates floral initiation and activates the floral homeotic genes^2^. As expected, *PtLFY* was up-regulated from floral induction to initiation and then down-regulated (Fig. 6). Previous study showed that *PTLF* is strongly expressed in developing inflorescences but is also detected in leaf primordia, young leaves, apical vegetative buds, and seedlings^10^. When overexpressing *LFY* constitutively in hybrid aspen, transgenic plants flowered *in vitro* within 7 months^68^, although this effect was later shown to be highly variable between poplar clones. The overexpression of *LFY* induces solitary flowers in male aspen clones; however, this occurs only infrequently in female clones^10^. As *PTLF* was less effective in accelerating flowering, the orthologous proteins may differ in their activities or regulatory interactions^8^. Interestingly, the catkins of *PtLFY*-RNAi poplars were small and lacked stigmas or ovules, while the trees had normal or robust vegetative growth^69^. *PtLFY* was also increased, along with that of *PtSOC1*, during dormancy release (Figs 4, 6). *Arabidopsis SOC1* forms a positive feedback loop with *AGL24*. These two factors may form a complex that stimulates the up-regulation of *LFY* ^2^.

*WUSCHEL* (*WUS*)-related homeobox (*WOX*) family genes play important roles in the maintenance and proliferation of the stem cell population in the shoot apical meristem; they also mediate floral organ development^70^. We identified four *PtWUS* and *PtWOX* genes; their expression patterns are shown in Fig. 6. In *Arabidopsis* floral meristem, *WUS* promotes the expression of *AG*, which then negatively feeds back on *WUS*, resulting in the down-regulation of stem cell proliferation and the promotion of determinacy^71^.

In *Arabidopsis*, *CO* initiates flowering via the up-regulation of *FT* ^17^. Similar to the *Arabidopsis CO*/*FT* regulon, the poplar *CO2*/*FT1* regulon controls the timing of flowering and regulates both SD-induced growth cessation and fall bud set^13^. However, the overexpression of *PtCO1*&*2* does not alter normal reproductive onset, spring bud break, or bud set in poplar^11,17^, although poplar *CO1* could rescue the late-flowering phenotype of *Arabidopsis co-1* mutants^17^. These results indicate that CO/FT was modified in poplar following the divergence of the *Arabidopsis* and poplar lineages. In our study, *PtCO1* transcript was most abundant in stage 8. Hsu *et al*.^17^ also found that poplar *CO1* was consistently expressed in reproductive buds, with the highest level in late winter (Fig. 6).

Members of the *FT*/*TFL1* family are important regulators of flowering time and dormancy^21,72^. We identified four *FT*/*TFL*1 genes in our floral transcriptome (Fig. 6B). *PtFT1* was up-regulated in the later stages of floral bud development, especially in response to winter temperatures, while *PtCEN1* was mainly expressed in the floral induction stage, decreasing thereafter (Fig. 6A,C). In *Arabidopsis*, *FT* promotes flowering, while *TFL1* represses its onset^72^. In *Populus*, the overexpression of *PtFT1*&*2* induces early flowering^13,14^. *PtFT1* acts as a strong promoter of precocious flowering and induces the formation of wild-type inflorescences^13^, whereas *PtFT2* induces only the formation of individual flowers^14^. Subsequent studies showed that sub-functionalization of these two genes had occurred, they have distinct seasonal expression patterns^15^. *PtFT1* is up-regulated in response to winter temperatures and determines reproductive onset, while *PtFT2* is up-regulated in response to the long-days and warm temperatures of the growing season and promotes vegetative growth, as well as the inhibition of bud set^13,15,16^. In our study, the pattern of *PtFT1* expression was consistent with those results (Fig. 6C). In *Arabidopsis*, *SVP* represses the expression of *FT*; likewise, our data showed the reciprocal expression of *PtSVP* and *PtFT* displayed (Figs 4 and 6). A reciprocal pattern of expression was also described for *DAM* and *FT* in leafy spurge^45^ and apple^25^. The down-regulation of *PopCEN1*&*2* by RNAi accelerated the onset of mature tree characteristics and regulated axillary meristem identity^18^. In our study, *PtCEN1* declined sharply from floral induction to initiation (Fig. 6C). Its low-level expression may allow the accumulation of transcripts of other floral promoters, resulting in floral initiation. Similar expression patterns were determined in the floral buds of apple^40^. Previous study showed that in *PopCEN1*-RNAi plants, the axillary inflorescences contained fewer flowers than did the wild-type, suggesting a role for *PopCEN1* in maintaining the indeterminacy of the inflorescence apex. *PopCEN1* also regulates the dormancy transition in *Populus*, while its overexpression alteres the chilling requirements and delays bud break^18^.

### Co-expression networks

**Figure 7:**
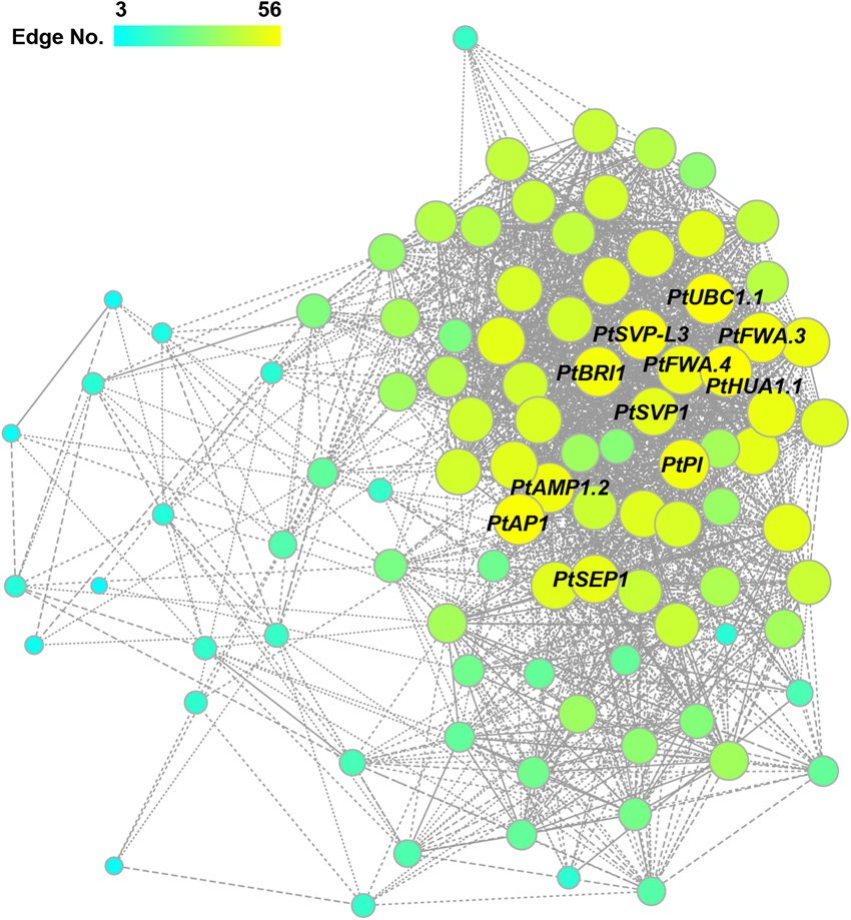
Co-expression network of 98 floral-related genes associated with flower meristem identity and flower development in *P*. *tomentosa*. The threshold of the Pearson correlation coefficient was set at ≥0.7 (or ≤−0.7). The larger and more yellow the circles, the higher the connectivity.

To search for the regulatory genes that participate in flowering in poplar, we selected 98 floral-related genes involved in flower meristem identity and flower development and used WGCNA to construct co-expression networks, in which the hub genes showed the densest connections (Fig. 7, Table S1). Many of the hub genes were MADS-box genes, including *PtAP1*, *PtSVP-L3*, *PtSEP1*, *PtPI*, and *PtSVP1*. In addition to *PtAP1*, the hub genes with the highest edge numbers were *PtHUA1*.*1* and *PtFWA*.4. In *Arabidopsis*, *HUA*1 is required for floral determinacy and it facilitates AG pre-mRNA processing^71^. *FLOWERING WAGENINGEN* (*FWA*) encodes a homeodomain-containing TF that controls flowering^73^. Other highly connected hub genes were *PtBRI1*, *PtUBC1*.*1*, and *PtAMP1*.2. In *Arabidopsis*, BRASSINOSTEROID INSENSITIVE 1 (BRI1) is involved in tapetum cell differentiation in the anther wall, pollen exine formation, and the positive regulation of flower development^74^. UBIQUITIN CARRIER PROTEIN 1 (UBC1) is associated with the negative regulation of flower development^75^. ALTERED MERISTEM PROGRAM 1 (AMP1) alters flowering time and photomorphogenesis and increases cytokinin biosynthesis^76^.

## Materials and Methods

### Plant material and RNA extraction

Adult male *P*. *tomentosa* trees were grown in the nursery of Beijing Forestry University (Beijing, China) in a natural environment. The axillary floral buds were collected from June 2013 through February 2014, to cover eight representative stages of flower development including floral induction, floral initiation, and organ differentiation: stage 1, floral induction; stage 2, primordia formation; stage 3, organogenesis; stages 4, enlargement; stage 5, archespore formation; stages 6 and 7: dormancy; stage 8: microsporogenesis (Fig. 1). The collected samples were immediately frozen in liquid nitrogen and stored at −80 °C until further use. Total RNA was isolated from the pooled samples as described previously^20^. For every stage, equal amounts of total RNA from eight biological replicates were pooled to promote sample homogeneity and decrease bias. The quality and quantity of the total RNA samples was analyzed using a NanoDrop ND-1000 spectrophotometer (Thermo Fisher Scientific, Inc.) and an Agilent 2100 Bioanalyzer (Agilent Technologies, Santa Clara, CA, USA).

### Floral tissue microscopy

Histological sections were prepared by fixing the floral buds from the eight different developmental stages in formalin-acetic acid. The buds were then dehydrated in a graded ethanol series and embedded in paraplast. Serial sections were obtained using a Leica microtome, mounted on microscope slides, stained with 1% safranin, and observed with an Olympus BX-61 microscope (Olympus, Tokyo, Japan).

### RNA deep sequencing

RNA samples for cDNA libraries and RNA-seq were prepared using the Illumina kit according to the manufacturer’s protocol (Illumina, San Diego, CA, USA). The eight libraries were sequenced separately with the Illumina HiSeq^TM^ 2000 platform using paired-end technology. Contamination detection was assessed by comparing the randomly selected 500,000 reads from each raw sequencing dataset against the nucleotide database from NCBI. The files of raw fastq were also checked by FastQC. After removal of the adapter sequences, low-quality sequences and reads with >10% Q < 20 bases, all valid reads were pooled for *de novo* assembly using the Trinity program.

### Annotation of unigenes

The longest transcript at each locus was regarded as a unigene. To understand the functions of the identified unigenes, they were annotated using BLASTx alignment against the NCBI non-redundant protein (Nr), Swiss-Prot protein, and Cluster of Orthologous Groups (COG) databases with an E-value cut-off of 10^−5^ ^77^. Functional annotation by Gene Ontology (GO) terms was analyzed using Blast2GO^78^. Pathway annotation with the Kyoto Encyclopedia of Genes and Genomes (KEGG) was performed using the KAAS server^79^. Unigenes were also annotated against the TAIR10 database of *Arabidopsis* and the v3.0 database of *P*. *trichocarpa*.

### Differential gene expression and enrichment analysis

Gene expression levels were estimated as fragments per kilobase of transcript per million mapped reads (FPKM)^80^. Differentially expressed genes (DEGs) were analyzed using the DESeq software^81^. The false discovery rate (FDR) was used to determine the *p*-value threshold in multiple tests^82^. Due to the time-series parameters of this study, seven sets of DEG analysis were separately performed. In each analysis, a criteria of |log_2_(Ratio)| ≥ 1 and an FDR of ≤ 0.001 between the two consecutive time points was used to identify DEGs. GO enrichment was performed based on a *p*-value < 0.05, after applying the Benjamini Hochberg correction using agriGO^83^. Hierarchical and k-means clustering were generated by MultiExperiment Viewer (MeV; ver. 4.6.2). A principal component analysis (PCA) was performed using MeV.

### Quantitative reverse transcription PCR

Total RNA was treated with RQ1 DNase I (Promega, Madison, WI, USA) to remove contaminating genomic DNA. The first-strand cDNA was synthesized using 1 μg of RNA with oligo d(T)_20_ and a reverse transcription system (Promega). It was then diluted 1:10 with ddH_2_O and used as a template for RT-qPCR amplification on a 7500 Fast real-time PCR system platform (Applied Biosystems, Foster City, CA, USA) using SYBR^®^ Premix Ex Taq^TM^ (TaKaRa, Otsu, Japan). The gene-specific primers employed in the RT-qPCR analysis are listed in Table S2. Thermal cycling was performed at 95 °C for 30 s, followed by 40 cycles of amplification (95 °C for 5 s, 60 °C for 20 s, 72 °C for 15 s), with a final extension of 7 min at 72 °C. The plates were read every 0.2 °C for 1 s from 70 to 95 °C, to generate melting curves. *PtACTIN* ^19^ was used as the internal reference gene for normalization according to the 2^−ΔΔCt^ method. Each reaction was performed in three replicates.

### Annotation, phylogenetic analysis, and co-expression network of flower-related genes

Blastn searches of the Phytozome database, using *Arabidopsis* and *P*. *trichocarpa* genes as queries, were used to identify flower-related genes in *P*. *tomentosa*. Multiple sequence alignments and the phylogenetic analysis were performed as described previously^84^. Transcription factors (TFs) were identified by using the unigenes from the assembled sequences in a BLAST analysis against TFs from *P*. *trichocarpa* in the Plant Transcription Factor Database (PlantTFDB)^85^. A weighted genes co-expression network analysis (WGCNA) was performed using OmicsShare Tools.

## Electronic supplementary material

Supplementary information
