# Supplementary material for: A Global View of Transcriptome Dynamics During Male Floral Bud Development in Populus tomentosa
Source: Sci Rep. 2018 Jan 15;8:722. doi: 10.1038/s41598-017-18084-5 (PMC5768756; doi:10.1038/s41598-017-18084-5)
Supplement: Supplementary file 1 — Supplementary information [file 41598_2017_18084_MOESM1_ESM.pdf]

Supplementary Information

A Global View of Transcriptome Dynamics During Male Floral Bud Development in *Populus tomentosa*

Zhong Chen[1,2], Pian Rao[2], Xiaoyu Yang[2], Xiaoxing Su[3], Tianyun Zhao[2], Kai Gao[2], Xiong Yang[2] and Xinmin An[2,4]

[1]Key Laboratory of Silviculture and Conservation of the Ministry of Education, College of Forestry, Beijing Forestry University, Beijing, 100083, China

[2]National Engineering Laboratory for Tree Breeding, Key Laboratory of Genetics and Breeding in Forest Trees and Ornamental Plants of the Ministry of Education, College of Biological Sciences and Biotechnology, Beijing Forestry University, Beijing, 100083, China

[3]Berry Genomics Co., Ltd., Beijing, 100015, China

[4]Department of Forest Ecosystems and Society, Oregon State University, Corvallis, OR, 97331, USA

Zhong Chen, Pian Rao and Xiaoyu Yang contributed equally to this work.

Correspondence: Z. Chen or X. An

Email: zhongchen@bjfu.edu.cn or anxinmin@bjfu.edu.cn

Address: No.35 Qinghua East Road, Haidian District, P. O. Box 118, Beijing Forestry University, Beijing 100083, China

Supplementary Information

Figure S1. Length distribution of the transcripts and assembled unigenes.

Figure S2. Global analysis of the transcriptome datasets. **A**, Distribution of the top BLASTX hits for unigenes in the non-redundant protein (Nr) database. **B**, Cluster of Orthologous Groups (COG) classifications in *P. tomentosa*. **C**, Gene Ontology (GO) assignment of *P. tomentosa* unigenes.

Figure S3. Global view of the expressed genes. **A**, Principal components analysis of the eight samples in this study. **B**, Proportion of the expressed genes (based on fragments per kilobase of transcript per million mapped reads; FPKM) during the eight stages of flower development.

Figure S4. GO enrichment during flower development. The 20 significantly enriched GO (biological process, molecular function and cellular component) categories containing the up- and down-regulated genes of the eight stages of flower development.

Figure S5. GO enrichment during the various stages of flower development with respect to the preceding stage. Five significantly enriched biological process GO terms in the up-regulated genes during the various stages of flower development with respect to the preceding stage.

Figure S6. Expression patterns of differentially expressed transcription factors (TFs). **A**, The top 15 differentially expressed TFs during various stages of flower development. **B**, Hierarchical cluster analysis and **C**, K-means clustering of the differentially expressed TFs during the eight developmental stages. Black numbers on the top are the number of genes for each cluster; the red numbers are the cluster labels.

Figure S7. Expression profiles of MIKC* MADS-box genes in the floral transcriptome, and the identification of those genes by RT-qPCR. **A**, Heatmap

showing the expression patterns of the identified MIKC* MADS-box genes in the floral transcriptome of *P. tomentosa*. **B**, RT-qPCR validation of the MIKC* MADS-box gene expression levels in floral buds. The blue and pink lines were derived from the RNA-seq and RT-qPCR data, respectively.

Figure S8. Expression profiles of receptor-like kinase (RLK) genes in the floral transcriptome and their RT-qPCR validation. **A**, Hierarchical cluster analysis of the RLK genes expressed during the eight stages. **B**, RT-qPCR validation of the expression profiles obtained by RNA-seq.

Figure S9. Expression profiles of the genes involved in anther, tapetum, and pollen development. **A**, Heat map of gene expression. **B**, RT-qPCR confirmation of the expression profiles obtained by RNA-seq transcriptome analysis. The blue and pink lines were derived from the RNA-seq and RT-qPCR data, respectively. The values are means ± SD. ACOS5, ACYL-COA SYNTHASE; AMS, ABORTED MICROSPORES; CYP, CYTOCHROME P450; EMS1, EXCESS MICROSPOROCYTES1; βGLU, BETA-1,3-GLUCANASE; OLE, OLEOSIN; PME, PECTIN METHYLESTERASE; POE1, POLLEN OLE E 1 ALLERGEN AND EXTENSIN; SKU5, MONOCOPPER OXIDASE; TDF, DEFECTIVE IN MERISTEM DEVELOPMENT AND FUNCTION; TPD1, TAPETUM DETERMINANT1.

Table S1. Selected floral-related genes in co-expression network.

Table S2. Primer sequences used in the RT-qPCR analysis performed in this study.

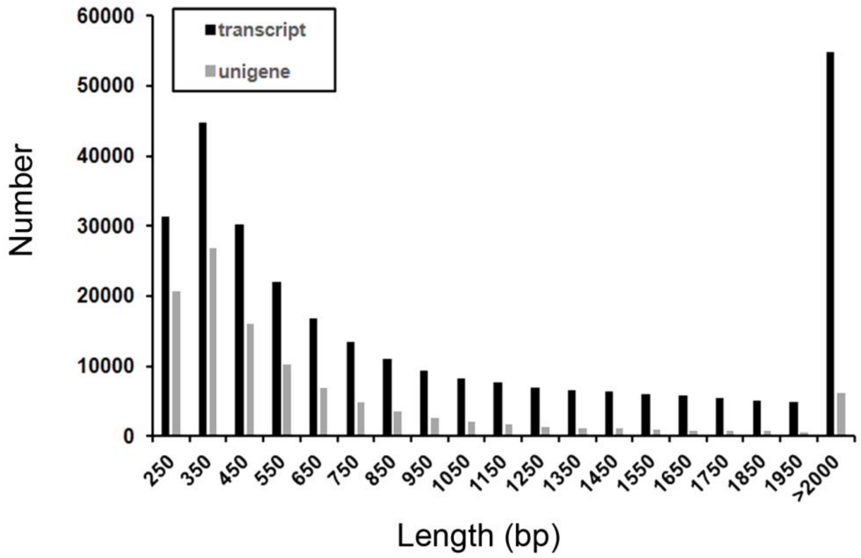

Figure S1. Length distribution of the transcripts and assembled unigenes.

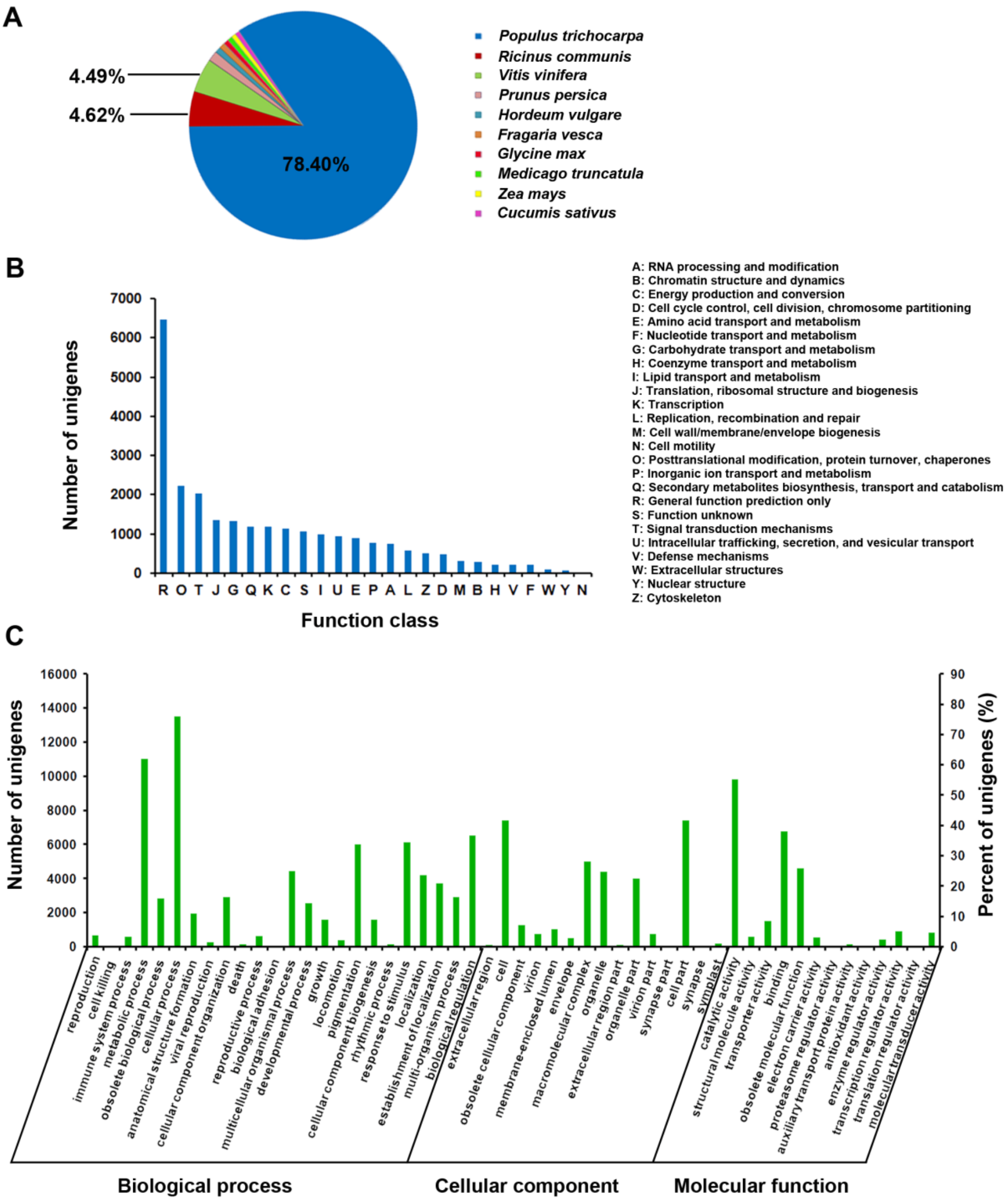

Figure S2. Global analysis of the transcriptome datasets. **A**, Distribution of the top BLASTX hits for unigenes in the non-redundant protein (Nr) database. **B**, Cluster of Orthologous Groups (COG) classifications in *P. tomentosa*. **C**, Gene Ontology (GO) assignment of *P. tomentosa* unigenes.

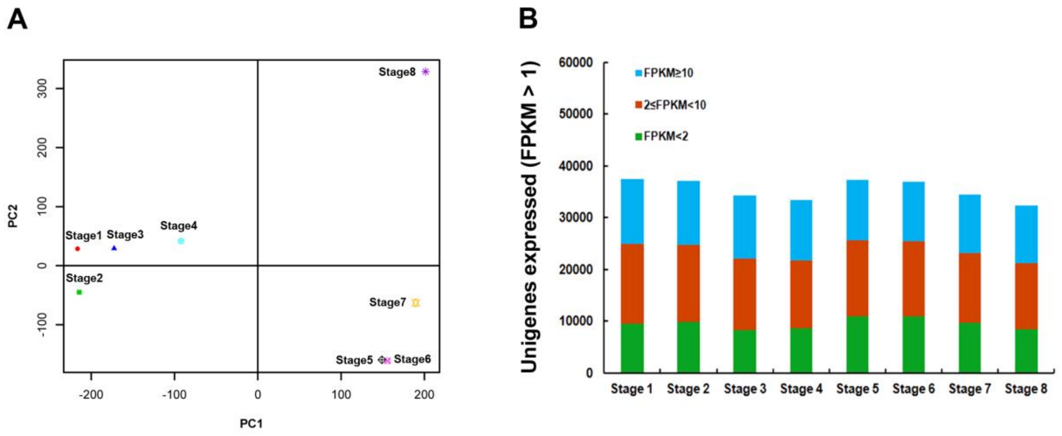

Figure S3. Global view of the expressed genes. **A**, Principal components analysis of the eight samples in this study. **B**, Proportion of the expressed genes (based on fragments per kilobase of transcript per million mapped reads; FPKM) during the eight stages of flower development.

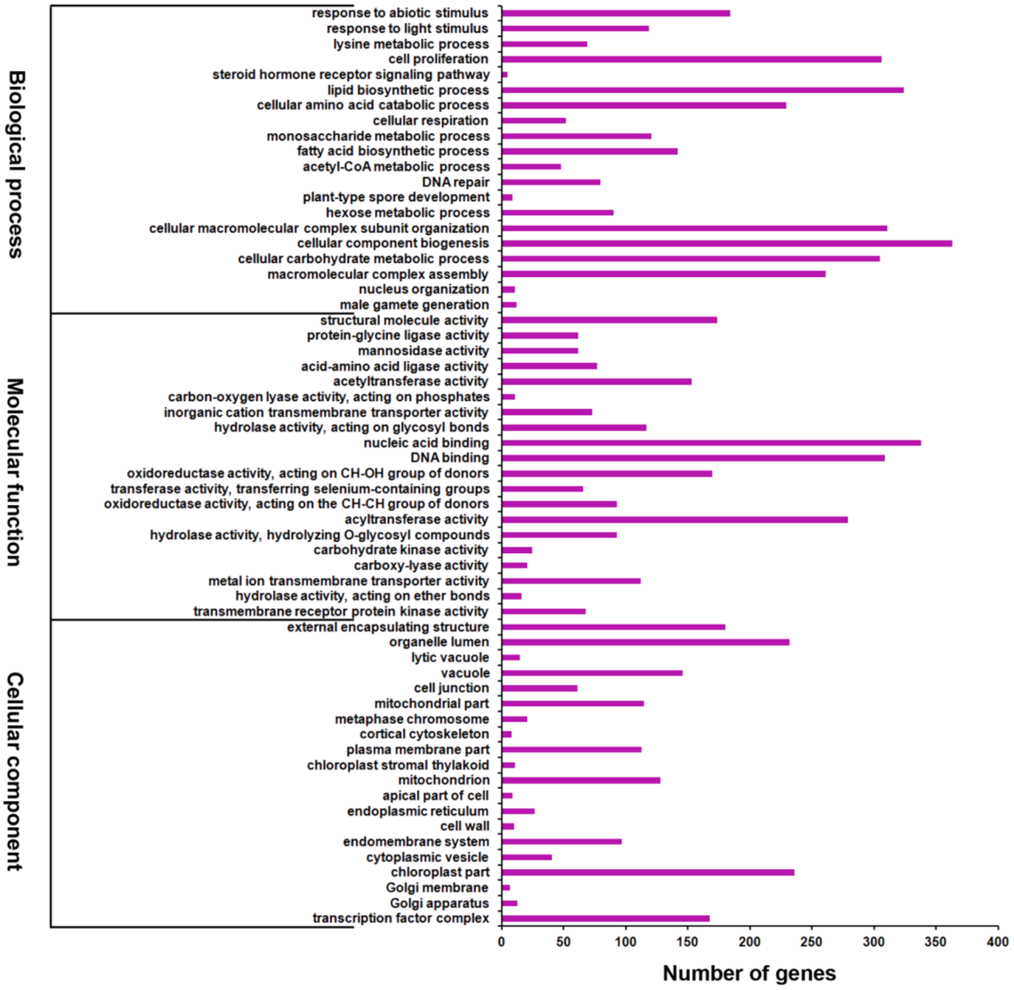

Figure S4. GO enrichment during flower development. The 20 significantly enriched GO (biological process, molecular function and cellular component) categories containing the up- and down-regulated genes of the eight stages of flower development.

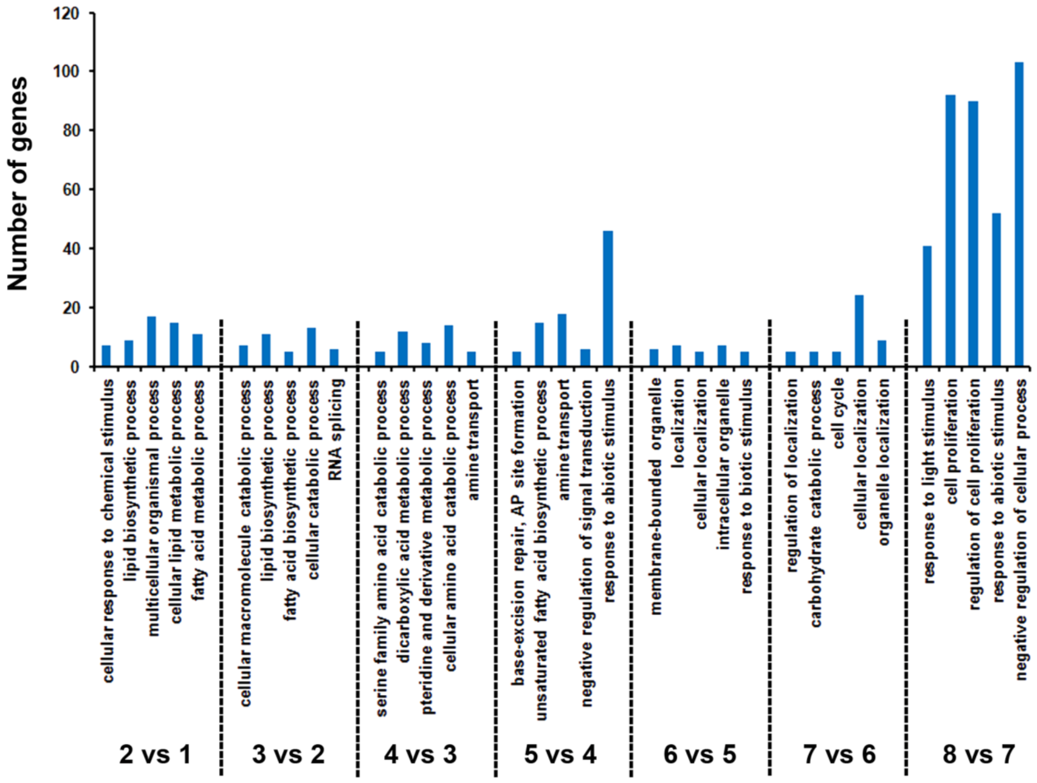

Figure S5. GO enrichment during the various stages of flower development with respect to the preceding stage. Five significantly enriched biological process GO terms in the up-regulated genes during the various stages of flower development with respect to the preceding stage.

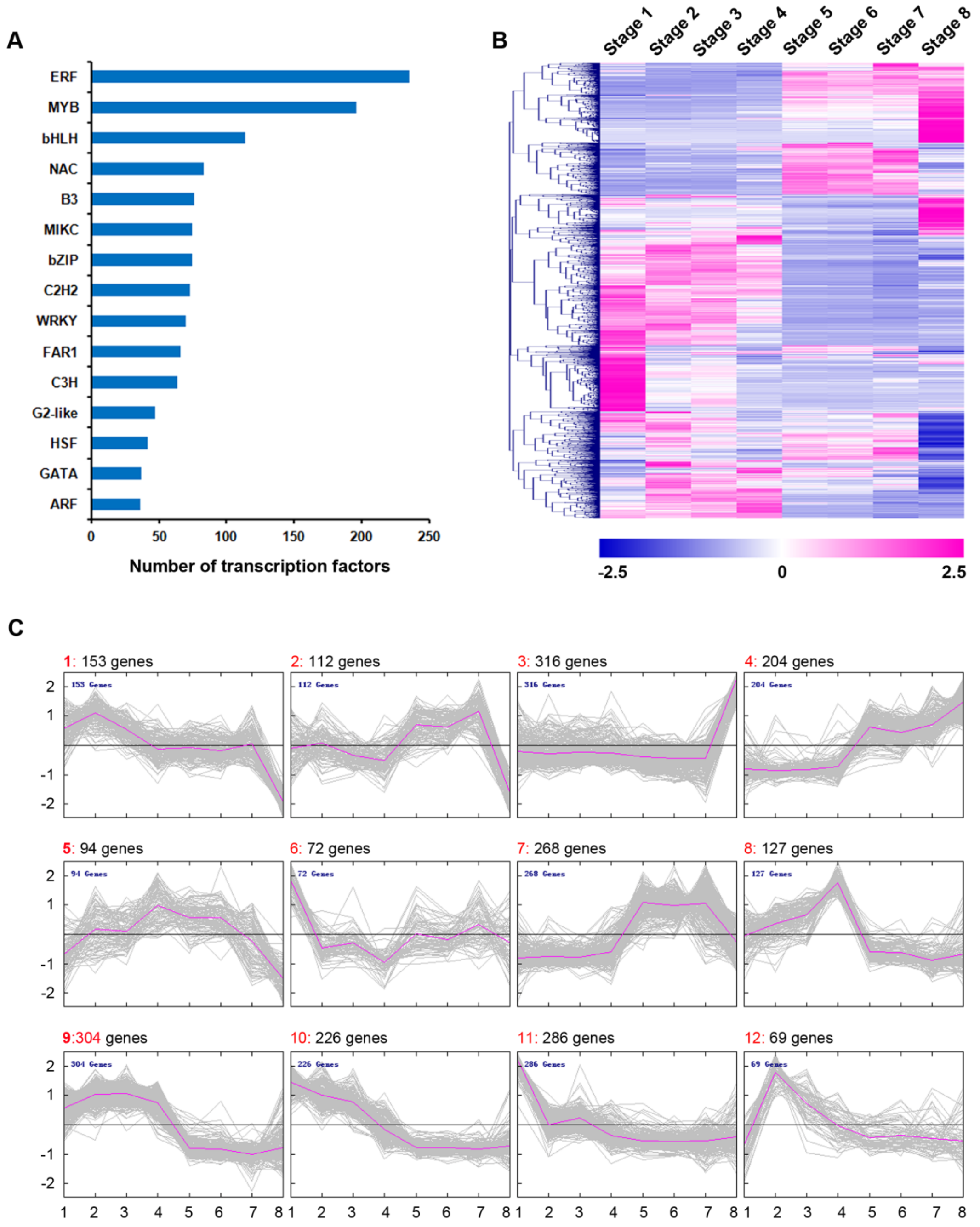

Figure S6. Expression patterns of differentially expressed transcription factors (TFs). **A**, The top 15 differentially expressed TFs during various stages of flower development. **B**, Hierarchical cluster analysis and **C**, K-means clustering of the differentially expressed TFs during the eight developmental stages. Black numbers on the top are the number of genes for each cluster; the red numbers are the cluster labels.

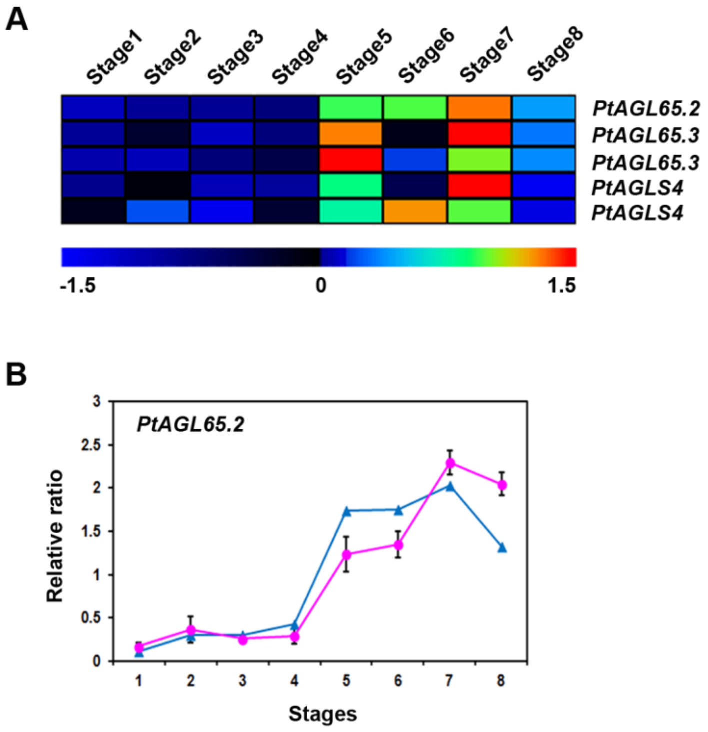

Figure S7. Expression profiles of MIKC* MADS-box genes in the floral transcriptome, and the identification of those genes by RT-qPCR. **A**, Heatmap showing the expression patterns of the identified MIKC* MADS-box genes in the floral transcriptome of *P. tomentosa*. **B**, RT-qPCR validation of the MIKC* MADS-box gene expression levels in floral buds. The blue and pink lines were derived from the RNA-seq and RT-qPCR data, respectively.

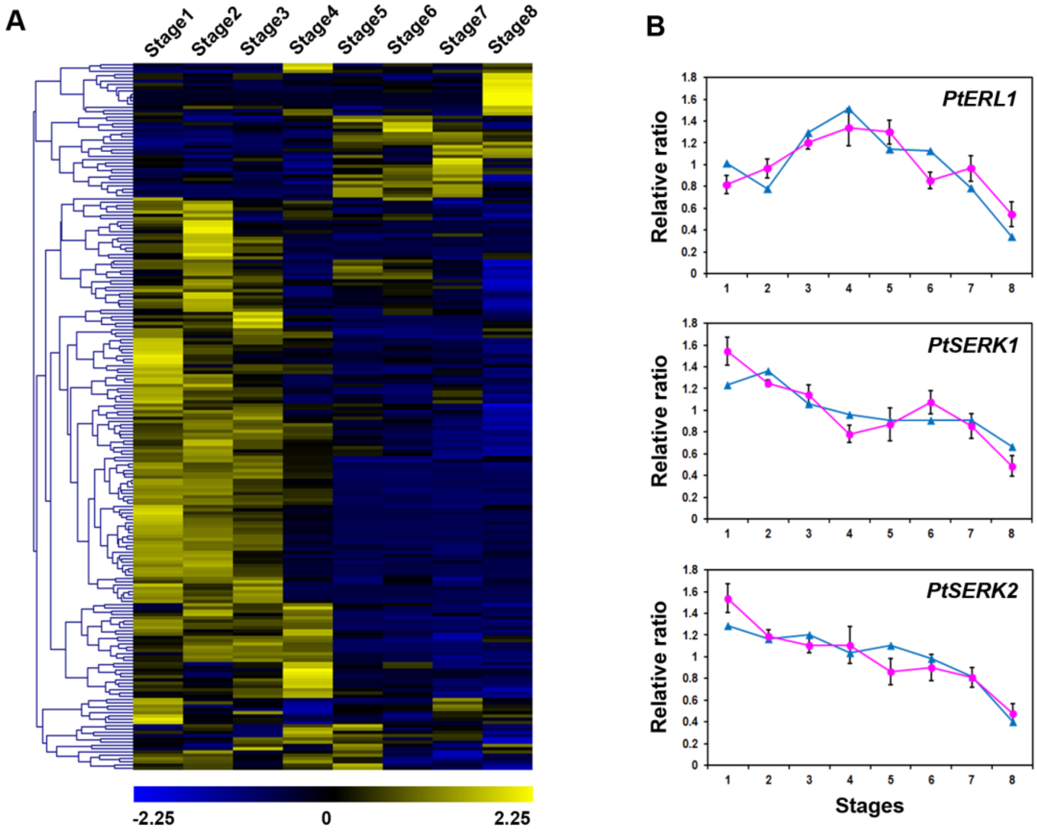

Figure S8. Expression profiles of receptor-like kinase (RLK) genes in the floral transcriptome and their RT-qPCR validation. **A**, Hierarchical cluster analysis of the RLK genes expressed during the eight stages. **B**, RT-qPCR validation of the expression profiles obtained by RNA-seq.

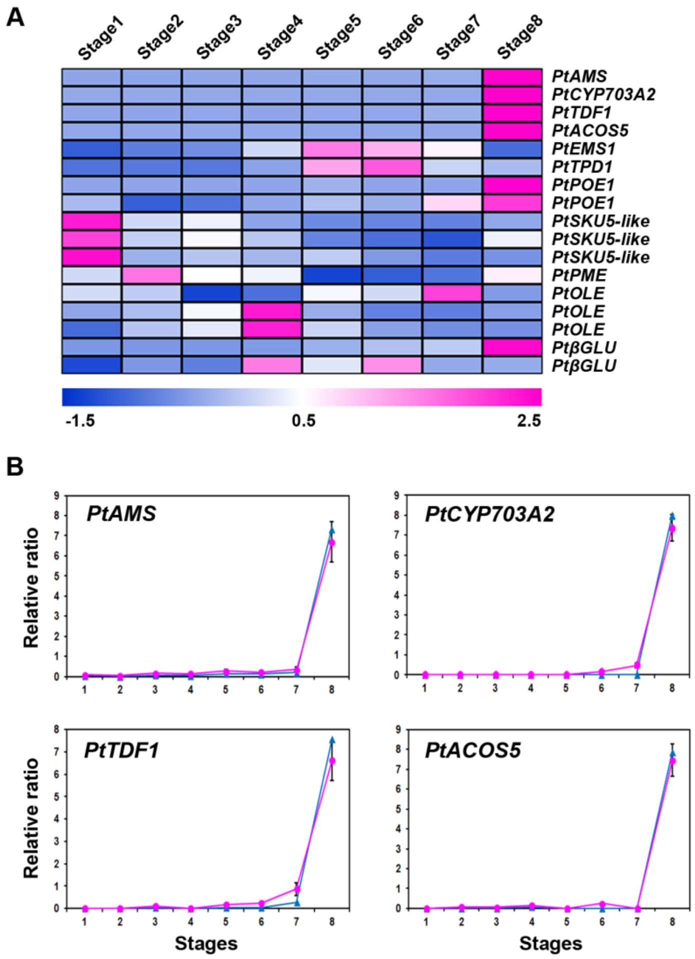

Figure S9. Expression profiles of the genes involved in anther, tapetum, and pollen development. **A**, Heat map of gene expression. **B**, RT-qPCR confirmation of the expression profiles obtained by RNA-seq transcriptome analysis. The blue and pink lines were derived from the RNA-seq and RT-qPCR data, respectively. The values are means ± SD. ACOS5, ACYL-COA SYNTHASE; AMS, ABORTED MICROSPORES; CYP, CYTOCHROME P450; EMS1, EXCESS MICROSPOROCYTES1; βGLU, BETA-1,3-GLUCANASE; OLE, OLEOSIN; PME, PECTIN METHYLESTERASE; POE1, POLLEN OLE E 1 ALLERGEN AND EXTENSIN; SKU5, MONOCOPPER OXIDASE; TDF, DEFECTIVE IN MERISTEM DEVELOPMENT AND FUNCTION; TPD1, TAPETUM DETERMINANT1.

Table S1 Selected floral-related genes in co-expression network.

Populus V3.0	gene name	unigene	stage 1	stage 2	stage 3	stage 4	stage 5	stage 6	stage 7	stage 8
Potri.016G064300	*AMP1.1*	comp645841_c0	2.14	2.22	2.1	1.43	2.35	1.73	2.05	2.61
Potri.006G153300	*AMP1.2*	comp646623_c0	3.44	3.37	3.16	3.43	1.41	1.65	1.82	0.69
Potri.018G128300	*ARP6*	comp650372_c1	14.56	12.86	13.04	12.64	14.08	13.04	11.29	9.01
Potri.005G195400	*ASHH1*	comp655703_c0	27.25	33.11	39.07	37.03	20.54	22.73	32.11	28.79
Potri.008G003200	*ATFYPP3*	comp652578_c1	81.24	78.56	73.51	80.85	123.6	109.9	125.6	106.89
Potri.001G244100	*ATVGT1.1*	comp645949_c0	25.61	32	29.64	34.67	22.37	18.78	18.33	11.49
Potri.019G055600	*ATVGT1.2*	comp648924_c2	18.53	21.79	19.49	18.27	31.3	31.94	36.05	23
Potri.002G232800	*ATX1*	comp655578_c0	41.29	25.2	27.11	16.29	15.05	17.58	17.07	11.49
Potri.002G001000	*ATXR7*	comp655771_c0	11.42	11.93	13.2	9.67	7.81	9.96	5.23	2.58
Potri.007G078100	*BIN1*	comp655678_c0	43.05	43.3	31.53	24.06	11.62	9.97	9.77	8.29
Potri.006G203000	*BLH1*	comp651968_c0	129.99	296.41	227.3	203.08	245.31	237.88	234.75	100.99
Potri.002G159900	*CRP*	comp655976_c0	33.32	39.48	33.27	27.13	26.39	25.39	27.83	5.77
Potri.001G420400	*DDL*	comp644312_c0	24.14	29.07	31.07	42.11	72	71.26	63.86	27.82
Potri.005G109500	*FDP*	comp655407_c0	71.67	59.18	51.67	38.03	51.06	46.02	46.77	26.07
Potri.014G075200	*FWA.1*	comp607810_c0	89.11	98.66	85.32	37.71	22.56	27.67	19.17	12.3
Potri.004G020400	*FWA.2*	comp650383_c0	35.51	44.83	45.37	27.28	9.61	9.29	11.45	7.56
Potri.015G034100	*FWA.3*	comp653619_c0	17.19	16.04	9.62	8.88	3.59	2.25	1.05	0.32
Potri.003G052400	*FWA.4*	comp653719_c1	64.55	75.81	73.36	51.89	41.92	35.49	26.4	20.89
Potri.015G141800	*FWA.5*	comp653737_c0	7.29	9.49	9.49	6.39	5.08	5.12	2.95	3.1
Potri.014G152000	*FWA.6*	comp656037_c0	57.33	70.87	64.26	61.26	76.6	72.58	77.71	54.1
Potri.006G201900	*HAP2B*	comp652355_c0	1.19	1.97	1.93	3.53	1.48	1.25	1.07	1.03
Potri.007G045300	*ICU2*	comp650780_c0	6.26	4.65	5.07	5.13	2.81	2.38	3.74	6.38
Potri.013G046700	*JMJ14.1*	comp595650_c0	11.06	8.37	6.93	3.97	6.98	5.97	7.18	4.05

Potri.002G037300	*JMJ14.2*	comp652984_c0	16.13	12.5	12.58	11.45	21.67	17.84	19.23	13.58
Potri.001G440900	*JMJ14.3*	comp654970_c0	4.09	2.51	2.29	2.74	4.49	2.81	4.15	2.14
Potri.008G073700	*LAC8.1*	comp634635_c0	19.27	13.89	17.52	19.42	6.9	6.89	3.37	4.49
Potri.013G152700	*LAC8.2*	comp648767_c0	70.74	49.66	72.64	77.6	8.88	7.19	5.93	3.92
Potri.011G127600	*LDL2*	comp649209_c0	10.52	10.64	10.3	9.47	10.35	8.1	9.19	5.21
Potri.002G013100	*LSD1*	comp650980_c0	11.5	8.81	8.26	7.8	10.37	8.74	10.78	6.7
Potri.010G227200	*LSD1.3*	comp655541_c0	14.94	16.8	15.39	14.44	12.69	12.31	10.5	5.03
Potri.005G071900	*LMI1*	comp653987_c1	609.43	865.1	667.28	544.86	175.87	179.41	129.93	315.22
Potri.004G126600	*MIF1*	comp596808_c0	3.55	2.12	2.03	0.95	0.13	0.24	0.06	0.56
Potri.015G103900	*MMP*	comp633745_c0	9.06	5.05	6.76	5.16	4.13	6.33	3.72	3.8
Potri.008G089000	*NAP*	comp632298_c0	0.56	5.21	1.39	5.17	78.36	78.53	114.98	27.58
Potri.004G020300	*PCFS4*	comp654344_c0	10.82	12.13	11.35	10.66	10.14	9.91	9.62	3.95
Potri.002G104000	*PGI*	comp646616_c0	52.84	37.37	37.5	35.18	49.28	45.92	52.18	42.52
Potri.001G207700	*PIE1*	comp655800_c0	10.18	10.24	8.85	7.84	8.99	8.26	7.31	1.89
Potri.004G213300	*PNF.1*	comp636783_c0	6.83	5.84	3.88	1.82	0.95	0.84	0.67	0.84
Potri.009G009900	*PNF.2*	comp652582_c0	31.21	30.91	30.48	19.55	6.33	6.59	6.76	7.85
Potri.007G032700	*PNY.1*	comp610306_c0	4.7	6.73	4.96	3.21	1.35	0.63	0.94	0.98
Potri.010G197300	*PNY.2*	comp640054_c0	18.4	18.57	20.34	19.2	6.69	6.23	5.42	9.74
Potri.009G120800	*PNY.3*	comp655040_c0	80.73	103.17	79.22	46.31	51.29	53.19	61.08	49.67
Potri.003G180100	*PRE*	comp654070_c0	12.31	11.49	8.88	8.58	16.1	16.27	17.53	6.29
Potri.004G064300	*AG1*	comp637467_c0	4.86	30.64	42.01	83.61	83.74	81.23	88.07	162.49
Potri.014G074100	*AGL6*	comp595601_c0	4.13	7.44	9.13	9.78	15.37	14.1	16.4	61.45
Potri.012G132600	*AGL6-L2*	comp608102_c1	10.79	17.7	20.64	24.65	4.16	4.49	2.56	7.17
Potri.010G154100	*AP1*	comp625282_c1	33.01	95.18	101.52	109.19	327.2	343.94	373.81	463.33
Potri.002G028400	*AP3.1*	comp618279_c0	2.07	15.69	20.34	32.71	31.09	28.56	28.05	53.09
Potri.007G017000	*AP3.2*	comp636167_c0	9.15	39.79	68.6	172.96	269.43	235.71	172.42	280.24

Potri.012G062300	*FUL*	comp599057_c0	19.19	44.24	42.75	45.15	45.59	48.92	56.43	166.86
Potri.004G115400	*FUL-L2*	comp646569_c0	16.08	15.9	19.15	14.68	15.01	12.74	11.3	34
Potri.015G106900	*LFY*	comp627910_c0	14.3	41.46	35.56	15.66	10.82	9.38	10.82	23.16
Potri.002G079000	*PI2*	comp651100_c1	15.75	75.66	117.24	250.07	306.35	293.51	291.34	370.83
Potri.001G328400	*SEP/AGL6-L*	comp619848_c0	0.15	0	0.29	0	6.63	5.13	14.67	5.77
Potri.004G115500	*SEP1*	comp636886_c0	13.98	42.5	59.88	119.16	213.79	198.91	232.18	545.65
Potri.008G098400	*SEP2*	comp617509_c0	27.22	61.91	60.65	87.18	44.54	44.42	53.03	158.95
Potri.001G058400	*SEP3*	comp623420_c0	12.77	44.12	56.84	61.73	97.65	95.55	113.99	223.07
Potri.019G077200	*STK2*	comp641584_c0	0.04	0.27	0.48	0.51	0.4	0.14	0.25	15.19
Potri.015G098400	*TM8*	comp627453_c0	31.66	42.34	25.7	13.07	5.48	6.67	3.16	21.03
Potri.005G114700	*WUSa*	comp625070_c0	0.3	0.86	4.54	12.13	4.65	4.95	1.21	3.53
Potri.001G225700	*RPI2.1*	comp607444_c0	3.01	0.9	0.93	2.32	3.02	1.83	2.88	5.1
Potri.010G115300	*RPI2.2*	comp641786_c0	57.99	16.85	18.93	14.46	22.18	21.7	18.83	18.59
Potri.005G052000	*RPI2.3*	comp644034_c0	26.53	20.97	17.49	24.75	17.22	13.86	10.92	21.66
Potri.013G152600	*SEF*	comp652963_c0	41.37	33.32	41.06	40.19	9.19	11.19	6.39	4.63
Potri.008G183000	*TEL1*	comp654460_c0	13.84	12.49	7.07	18	32.65	31.65	16.69	4.49
Potri.002G079100	*EFS.1*	comp656045_c0	13.21	11.55	9.67	6.96	9.41	8.54	10.23	3.88
Potri.005G182100	*EFS.2*	comp648623_c1	2.89	3.07	2.18	2.14	2.38	2.43	2.62	0.27
Potri.013G083800	*T1N6_2*	comp631491_c1	2.23	2.27	1.84	1.26	0.21	0.11	0	0.49
Potri.013G064400	*UBC1.1*	comp632489_c0	152.59	176.29	184.81	218.31	325.36	305.75	378.17	370.84
Potri.010G216200	*TOE1.2*	comp651523_c0	3.93	3.77	2.77	2.11	6.96	6.81	7.37	6.77
Potri.016G084500	*TOE1.1*	comp652358_c0	93.08	73.1	66.54	58.12	162.82	163.09	181.46	176.28
Potri.007G046200	*AP2.15*	comp650314_c0	32.68	52.5	51.26	51.8	46.49	51.24	45.92	35.16
Potri.010G216200	*SMZ.1*	comp651523_c0	3.93	3.77	2.77	2.11	6.96	6.81	7.37	6.77
Potri.016G084500	*SMZ.2*	comp652358_c0	93.08	73.1	66.54	58.12	162.82	163.09	181.46	176.28

Potri.007G046200	*SMZ.3*	comp650314_c0	32.68	52.5	51.26	51.8	46.49	51.24	45.92	35.16
Potri.008G045300	*SNZ.1*	comp651523_c0	3.93	3.77	2.77	2.11	6.96	6.81	7.37	6.77
Potri.016G084500	*SNZ.2*	comp652358_c0	93.08	73.1	66.54	58.12	162.82	163.09	181.46	176.28
Potri.005G092700	*HUA2*	comp655284_c0	10.69	17.23	11.39	14.28	14.83	14.08	16.43	6.97
Potri.010G176300	*HUA1.2*	comp649041_c1	87.93	104.08	89.88	73.53	72.33	69.9	73.34	61.24
Potri.008G080300	*HUA1.1*	comp639588_c0	72.03	67.87	66.82	54.02	40.83	43.6	34.66	34.89
Potri.017G042500	*HEN4*	comp653730_c1	11.63	11.07	7.83	6.14	6.96	6.46	8.05	4.36
Potri.013G145400	*ULT1*	comp651726_c0	48.22	44.04	44.72	35.33	72.51	56.41	51.95	87.68
Potri.008G118300	*PAN*	comp643218_c0	9.61	17.48	17.13	8.18	7.42	6.4	3.78	2.69
Potri.002G072900	*SEU*	comp643473_c0	25.37	36.4	24.02	25.71	34.45	30.33	33.87	6.08
Potri.006G248100	*LUG.1*	comp653250_c0	59.51	51.36	56.9	45.27	73.83	72.61	122.39	33.21
Potri.003G178600	*LUG.2*	comp655527_c0	58.85	62.6	58.4	53.43	50.8	52.71	55.85	37.8
Potri.002G241800	*LUG.3*	comp649498_c0	40.93	43.07	46.19	35.13	34.74	33.9	45.5	43.99
Potri.001G160900	*UFO*	comp651487_c0	5.09	8.76	6.66	3.09	1.59	0.84	0.81	1.33
Potri.004G203900	*CEN1*	comp627567_c0	13.44	1.94	0.33	0.06	0	0	0	0.04
Potri.007G010800	*SVP1*	comp633343_c1	45.87	29.73	34	21.41	7.19	5.13	5.16	7.26
Potri.005G155300	*SVP2*	comp636774_c0	87.33	49.27	43.37	24.88	4.18	5.9	2.98	4.49
Potri.007G115200	*SVP-L1*	comp648986_c0	153.77	121.16	87.59	65.42	31.22	35.99	29.97	23.71
Potri.017G044200	*SVP-L3*	comp633238_c1	62.46	65.5	46.34	32.71	8.46	7.52	6.35	6.33
Potri.007G115000	*SVP-L5*	comp625082_c0	13.34	5.96	3.65	4.26	2.57	2.39	2.13	3.58
Potri.014G074200	*SOC1*	comp629998_c0	161.2	98.88	82.11	52.69	62.14	63.64	44.61	113.94
Potri.003G119700	*SOC3.1*	comp636933_c2	28.68	21.26	17.42	14.62	13.43	15.34	17.58	14.9
Potri.001G112400	*SOC3.2*	comp641584_c0	0.04	0.27	0.48	0.51	0.04	0.14	0.25	15.19
Potri.008G077700	*FT1*	comp635896_c0	5.88	3.45	2.71	2.7	8.77	7.86	27.62	30.04

Table S2 Primer sequences used in the RT-qPCR analysis performed in this study.

Name	Unigene	Forward (5'-3')	Reverse (5'-3')
PtAGL65.2	comp648690_c1	CCTGCCCTTTGTGATCTCGT	CAGCGAGAAGAAGCCAGTGA
PtSOC1.1	comp629998_c0	ACACAAACAAGCAGCCTGTT	CCTGATTCTTTCTCGCACGG
PtFLC2	comp639876_c0	GTCTCAGTGTGCTTACCCCT	CTACCATGGGCCTGTGAAGT
PtFLC4	comp646395_c0	TGCACATATCACACGGCTTC	TCTTGCTGCCAAAGTTGCAT
PtSVP1	comp633343_c1	TGTGCAACTCAAATGGCCCC	GTTTCCCAGCGCTTGTCCAA
PtSVP2.2	comp636774_c0	GGAAAGAGACCTGCCCTTGTTG	TTCAGCATCAGATGGCCAACC
PtSVP-L1	comp648986_c0	GGAGAGAGACCGAGCACAAA	TCATCACTCTTTCTTCTCCTGCT
PtAP1.2	comp625282_c1	CCGCCCTTAAACACATTCGG	TGCGTCTGATACCATGGACC
PtFUL	comp599057_c0	TTCCTCAATTCCCGTTGCCA	AAAGAAGGACAAGGCGCTCC
PtAP3.1	comp618279_c0	CAACTGATATGCTGCGGACG	TGGGATGCCACTAACTTGCT
PtAP3.2	comp636167_c0	ATTGATCATCTGCGCGGTCT	TGCATAGAGGTTGGAAGCCC
PtPI2	comp651100_c1	CTGAGGCATCTGAAAGGGCA	GCACTCTCTTCCATGGCCAT
PtAG1	comp637467_c0	CCAGCTTCTCCGAGCAAAGA	CGAGAAACTGCTGCACTTGG
PtSTK2	comp641584_c0	GTACGCCAACAACAACAGCA	CAGCATCGCCCATTAAGTGC
PtSEP1.1	comp636886_c0	AGCTCCACTCTACCTCTCCC	TGGTGGGTGTTTCTCTCTCTC
PtSEP3.1	comp623420_c0	GCGTACTCCACATCTTCTGCA	TTGGCCCTCTAAGCAGCAAAG
PtSPL9	comp653646_c0	TGGAGCTGGAAATTGGGCAT	CCTTCCCCTGCTTCACCATT
PtSPL12	comp649925_c0	ATGGATTGAGCAGGCACCAA	GCCGTGCAAATGGAGGAATG
PtSPL16	comp615410_c0	ACGGGAACAAAGGGAGCTTT	TATTGCTGGAGGGCTAGGGT
PtSPL29	comp648892_c0	TGCACTGTTGGCAAAATCGG	CCAGCACTGCCAAATCATCG
PtAMS	comp645514_c0	GCACCGACGAAACCAAAACA	TCGTTGCAATCCGAGTGTGA
PtCYP703A2	comp642924_c0	GTACCAGAGTCACGCCAAGT	ATCAACACTCATGGGCTGGG
PtTDF1	comp625870_c0	ACCAACTCTTCAACCATGGCA	CCAAGATGCGGTCAACAAAGG
PtACOS5	comp629367_c0	AGCGAGTCCCCATCTTGTCTT	AGATCCCAGCTGCTTGTGTTG
PtERL1	comp655599_c0	GGCATTCCCAATTCCCTCCA	GGGGTTTGTTGCGTTGTCTC

PtSERK1	comp642221_c0	GATCTCCACCATTTGCCCCA	TGGGTCCTCTTCAGCAGGTA
PtSERK2	comp608894_c0	CCTTACTGTTTGCTGCCCCT	AGCCACCAATGAGCCATCTG
PtLFY	comp627910_c0	GCTTTCACGGCGAGTTTGTT	TTTGCTGCCGTGTAGTACCT
PtWUSa	comp625070_c0	AATGGTGCGGTTACTGTTGGG	ATCTTTTCAGCGGCTTCCTGG
PtWOX13a	comp648607_c0	TTGCTCCATCTGCCAGTACG	ATCAGCCGTAGTGCCAAACA
PtCO1	comp639018_c0	AATCAGCCCGGCAGTAAACA	GCCTCCTCTTTCTTGCACCT
PtFT1	comp635896_c0	TTGGCCATGAAACTGTGTGCT	ACTCTCCCTCTGGCAGTTGAA
PtCEN1	comp627567_c0	TCAACGTTTCCTTGCAGCTGT	GGAAGGGAGGTGGTGAGCTAT
Populus ACTIN		GAGGGAAGCCAAGATAGAGC	CGGAATCCACGAGACTACATAC
